# A study of steady-state strategies for collaborative quality improvement in new retail service supply chains considering emotional factors

**DOI:** 10.1371/journal.pone.0294175

**Published:** 2023-11-15

**Authors:** Bang Guo, Yixin Li

**Affiliations:** 1 Academy of Social Sciences, The Chinese University of Hong Kong, Hong Kong, 999077, China; 2 School of Management, Xi’an University of Science and Technology, Xi’an, 710054, China; Xuzhou University of Technology, CHINA

## Abstract

How to improve the quality of the new retail service supply chain (RSSC) has become a hot topic for enterprises and consumers. Considering the influence of the new RSSC enterprises’ emotional attitude on the decision-making of quality improvement, the theory of rank-dependent expected utility (RDEU) is combined with an evolutionary game, constructing an evolutionary game model of collaborative quality improvement of new RSSC, and analyzing the game strategy choice of each participant in collaborative quality improvement of new RSSC. The study shows that when only one party is emotional, the rationality of retail service integrators will promote the synergistic improvement of the quality of the new RSSC more than functional service providers. Moreover, pessimism and optimism have an inverted U-shaped effect on quality decisions. When both parties have emotions, functional service providers remain optimistic, and retail service integrators remain pessimistic or rational can promote the collaborative improvement of new RSSC quality. In addition, the effects of quality preference, peer mechanism, feedback mechanism, and risk mechanism on the collaborative quality improvement of new RSSC are analyzed. Based on the research findings, relevant countermeasures are proposed to incentivize new retailers to conduct collaborative improvement in quality in terms of establishing an open mechanism for negotiation and consultation, strengthening the emotion management of new retail node firms, and adjusting the transmission of quality signals, with a view to realizing the quality collaborative improvement of the new RSSC.

## 1. Introduction

The development of digital technologies [1–3], such as big data, cloud computing, artificial intelligence, blockchain, and meta-universe, has provided inexhaustible power for the transformation and upgrading of the retail industry [4]. New retail with digitalization as the core driving force [5–7] is continuously driving the reconstruction of retail and supply chain [8,9]. New RSSC operation management involves the coordination and optimization of multi-stage, multi-channel, and multi-interested subjects [10], and the relationship between the elements of the new retail business model and the scene elements is more dynamic [11], and how to improve the quality of service under a more complex operating environment has become the key to maintain the competitive advantage of RSSC enterprises [12].

To solve the quality problems of new RSSC due to the poor connection of nodal enterprises and the product quality level cannot meet the quality requirements of the market [13], many scholars have conducted research into the directions of product supply chain service quality, service supply chain service quality, and supply chain quality risk. For example, Reyniers *et al*. [14] found that the quality of supplier quality selection link, manufacturer review product link and the quality of generated products are affected by contractual factors such as the cost of guarantee in the after-sales service process. Seth *et al*. [15] elucidated the significance of the service quality that cannot be ignored in supply chain activities, and constructed a qualitative model with the help of the GAP (Geometry, Architecture, Process) methodology to assess the quality of the service. Liu *et al*. [16] studied the coordination mechanism of the logistics service supply chain from the perspective of the customer experience level by establishing a Stackelberg game model, and found that the perfect coordination of the logistics service supply chain can be realized by adopting quality supervision and repurchase contract at the same time. Ju *et al*. [17] take logistics service supply chain as the research object and focus on the relationship between integration quality, value co-creation and logistics service supply chain resilience, and find that digital technology has a positive moderating effect on the relationship between integration quality, value co-creation and resilience. Ouyang *et al*. [18] studied the optimal quality behaviors, optimal benefits, and the combination of conditions and strategies of the participating members of the retail service supply chain (RSSC) by comparing and contrasting them, and found that the ratio of benefit distribution and the distribution of quality costs determine the optimal quality behaviors of RSSC participating members, and become key factors for the participating members to choose the collaboration mode in the case of quality integration. Ren *et al*. [19] considered an IT service supply chain with software developers, service providers and customer firms, and found that when developers have fairness concerns or providers have fairness concerns, fairness concerns have a strong negative effect on the quality of the presale service, but a weaker negative effect on the price of the software and the price of the EWS.

Generally speaking, the relevant research is abundant, but there are still two shortcomings therein: most of the existing studies focus on the quality decision-making of a single enterprise, and less consider the joint decision-making among multiple enterprises; secondly, most of the existing studies focus on mechanism design and technical scheme introduction, and few studies discuss the influences of emotional factors of decision-makers on quality decision-making. Therefore, the rank-dependent expected utility (RDEU) theory is combined with evolutionary game theory, to construct an evolutionary game model of collaborative quality improvement in new RSSC, and analyze the circumstances under which the influence of decision makers’ emotions on quality decision-making is promoted and suppressed: from the perspective of emotional management, this provides countermeasures and suggestions for improving the collaborative quality of the new RSSC.

## 2. Literature review

### 2.1. Quality collaborative improvement

With the arrival of the new retail era, the consumer structure is constantly changing and upgrading, the consumer’s cognition of the new RSSC services is no longer limited to the unilateral cognition of a node, but to the cognition of the entire service chain of the new retail, and the consumer’s quality demand is also constantly upgraded from the traditional demand for the quality of commodities to the demand for the quality of the service of the entire supply chain, imposing more onerous requirements upon the supply side to improve the supply capacity and service quality[20]. Quality management is the core content of supply chain synergy and integration, and the quality of products/services in the market is not determined not only by the quality efforts of individual enterprises, but also relies on the joint efforts of the participating entities of the whole chain [21]. The improvement of the quality level of the new RSSC requires the supply chain node enterprises to agree from the idea of collaborative symbiosis. Coordinating the relationship between the entities and synergistically optimizing the resources owned by each entity is the basic guarantee to ensure the smooth operation of the operation chain [22]. Comprehensive new RSSC in the interconnection of consumers, retailers and producers as well as the synergistic characteristics of each link [18], how to incentivize the cooperative parties based on the quality of service to establish a stable, orderly, continuous, innovative dynamic equilibrium state, and constantly, through the quality of synergistic enhancement, stabilize the quality of the supply capacity [23], building a service supply chain with efficient operation and stable quality [24–26] has become a key issue to enhance the endogenous driving force of new retail development.

### 2.2. Evolutionary game

The “new retail” service supply chain is shaped as a multi-scenario, synergistic and integrated ecosystem, and the new retail operating model is driven by the improvement of service quality in the retail market, but there are significant dynamics in the service supply chain system in which quality synergistic behaviors are often uncoordinated due to the lack of coherent cooperation among multiple actors [27]. Evolutionary game theory has been widely used in many fields such as energy structure transformation [28], new energy industry development [29,30], dynamic allocation of medical resources [31], sustainable operation of PPP projects [32], carbon emission regulation [33], product quality regulation [34], etc. Due to its ability to characterize the dynamic process of adaptive adjustment of decision-making subjects’ strategies over time, it provides a systematic and effective research framework for the study of quality collaborative improvements [35]. Ma *et al*. [36] conducted a game analysis of quality strategies in a product-service supply chain by considering the reference effect and altruism, and found that the consumer’s reference effect induces an “anchoring state of mind” among consumers, which leads manufacturers to lower the level of quality and retailers to lower the level of service. Zhang *et al*. [37] explored the change trend of service quality effort through a game by considering the impact of quality preference on quality effort decision-making in a service supply chain. Zhang *et al*. [38] conducted a game analysis on how the government reward and punishment mechanism affects quality improvement in a shared manufacturing supply chain, and found that the dynamic reward and punishment mechanism promotes the collaborative improvement of quality in a shared manufacturing supply chain more than other mechanisms.

### 2.3. RDEU theory

Many scholars have found that decision makers have different emotional attitudes due to their differences in values and interests [39,40], and their information processing, value perception, and behavioral decisions are largely influenced and guided by their emotions and moods [41,42], and decision makers’ moods have a significant impact on the operation of the system [43]. Due to its own limitations, the traditional game analysis method cannot explain the influence of heterogeneous mood of decision makers on the evolution of behavior, which in turn makes the scientific and rigorous interpretation of the game results to be improved. Under the background of information asymmetry [44], as a finite rational individual [45], the strategy choice of each participant in the new RSSC is not always theoretically optimal, and their subjective emotions towards collaborative quality improvement profoundly affect the efficacy thereof, making it is necessary to consider the emotions of the participants in the new RSSC into the cooperative quality improvement game model.

RDEU theory considers the influence of participants’ emotions on decision-making behavior and incorporates subjective emotion indicators into the modelling process, which makes up for the shortcomings of the traditional game theory that does not consider enough of the real emotions [46]. Liu *et al*. [47] used RDEU game to analyze the equilibrium strategies of emitting countries and stakeholder countries in different cases, and found that the most expected emotional state of the country that prevents the emitting country from discharging nuclear wastewater is to maintain pessimism for stakeholder countries. Zhao *et al*. [48] constructed a neighborhood conflict game model under the influences of emotions by using RDEU theory and emotion function, and found that the government’s emotions have a greater effect on the evolutionary consequences compared with the people’s emotions. Liu *et al*. [49] combined the iron ore trade conflict between Chinese steel companies and Australian iron ore mining companies with the asymmetric hawk-dove game model to analyze the iron ore trade conflict between Chinese steel companies and Australian iron ore mining companies, and found that, when the emotional factors and asymmetric factors are equal, the probability of the Australian companies’ adopting the extreme antagonistic tactics is higher compared with that of the Chinese companies. Li *et al*. [50] studied the one-cycle newsboy problem in which the risk-averse newsboy with grade-dependent expected utility determines the optimal sales order of newspapers. The study found that compared with the expected utility newsboy model, risk aversion had a more significant impact on the optimal order quantity under RDEU model, and gave a closed-form solution of the dual utility theory. These studies have drawn many valuable conclusions about emotions, but there is very limited research on how emotional factors affect quality decisions.

## 3. Game model construction

### 3.1. Research framework

**Fig 1:**
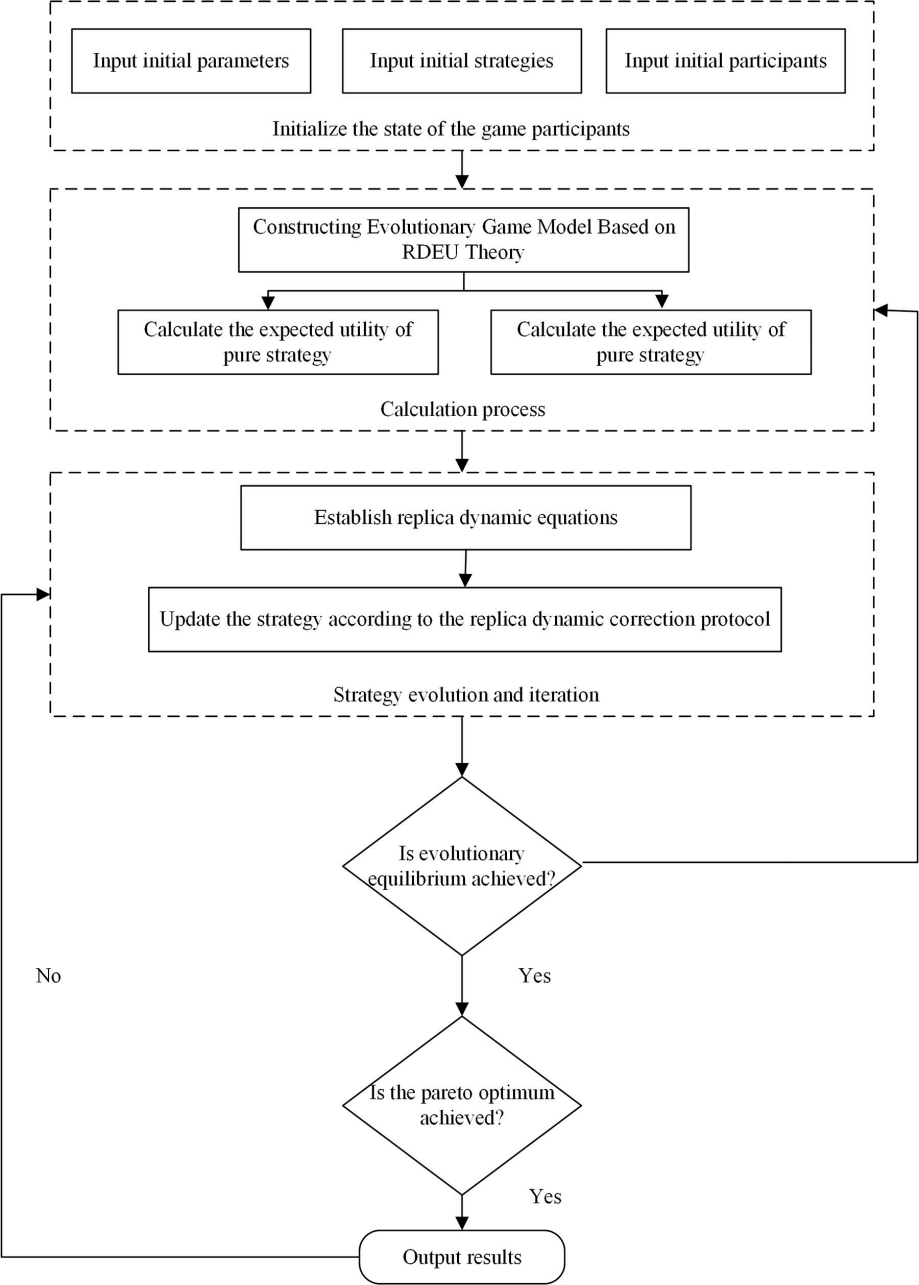
Research framework.

The aforementioned analysis theoretically clarifies the key problems to be solved in the present work. Here, the combination of RDEU theory to expand the evolutionary game model in a non-linear manner is discussed, along with the mechanism of influence of emotion on quality of decision-making. The research framework is shown in Fig 1.

### 3.2. Methodology

RDEU theory, first proposed by Quiggin [51], is a utility theory that takes into account the psychological preferences and emotions of decision makers. Under the conditions of decision uncertainty as well as high randomness, a real-valued function *C* defined by a utility function *U*(*x*) and a decision weight function *π*(*x*_*i*_) is used to represent the degree of decision makers’ preferences for different decisions, *i*.*e*., $V(x,u,\pi )=\sum_{i=1}^{n}\pi (x_{i})U(x_{i}),i=1,2,3,\cdots ,n$.

For the set of strategies *X* = {*x*_*i*_;*i* = 1,2,⋯*n*}, *P* = {*X* = *x*_*i*_} = *p*_*i*_. Assuming that strategy *x*_*i*_ is ranked according to the size of utility function *U*(*x*) and specifying *x*_1_>*x*_2_>⋯>*x*_*n*_, the utility rank of strategy *x*_*i*_ is defined as *RP*_*i*_. The probability distribution function of the strategy is therefore $RP_{i}=P(X\leq x_{i})=\sum_{\tau \geq i}^{n}p_{i},i=1,2,\cdots n$. Therefore, the larger the utility of the strategy, the larger its cumulative probability, and accordingly the greater the weight of the utility of the strategy in the decision.

The decision weight function is expressed as $\pi (x)=\omega (p_{i}+1-RP_{i})-\omega (1-RP_{i})$. Where *ω*(•) is a sentiment function and *ω*(•) is a monotonically increasing function that satisfies *ω*(0) = 0, *ω*(1) = 1.

The function *ω*(•) can be used to amplify or reduce the probability of *X*≤*x*.

There are three scenarios as follows:

1. when *ω*(*p*)<*p*, *ω*(•) is a concave function and *ω*(•) will reduce the likelihood of *X*≤*x*, indicating the pessimism of the participant;
2. When *ω*(*p*)>*p*, *ω*(•) is a convex function and *ω*(•) will magnify the likelihood of *X*≤*x*, indicating the optimism of the participant;
3. When *ω*(*p*) = *p*, the likelihood of *X*≤*x* is neither enlarged nor reduced, indicating the rationality of the participants.

**Fig 2:**
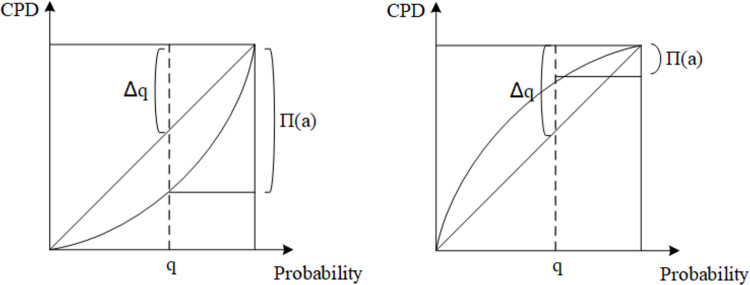
Utility function diagram.

As shown in Fig 2, CPD denotes the cumulative probability distribution, Fig 2(A) shows the utility function graph of the decision maker in the pessimistic case, and Fig 2(B) shows the utility function graph of the decision maker in the optimistic case.

The RDEU theory deals with decision weights in a non-linear manner by extending the utility theory in traditional game theory in a non-linear way. The method is able to portray the emotions of game participants positively under uncertain conditions in both theory and practice, therefore, the RDEU theory can overcome the shortcomings of traditional game theory in the attitudinal dimension to a certain extent, and the evolutionary game analysis that incorporates the theory is also able to more objectively and accurately portray the emotional state of each participant in shared manufacturing and the influence of their emotional intensity on behavioral decisions.

### 3.3. Problem description

**Fig 3:**
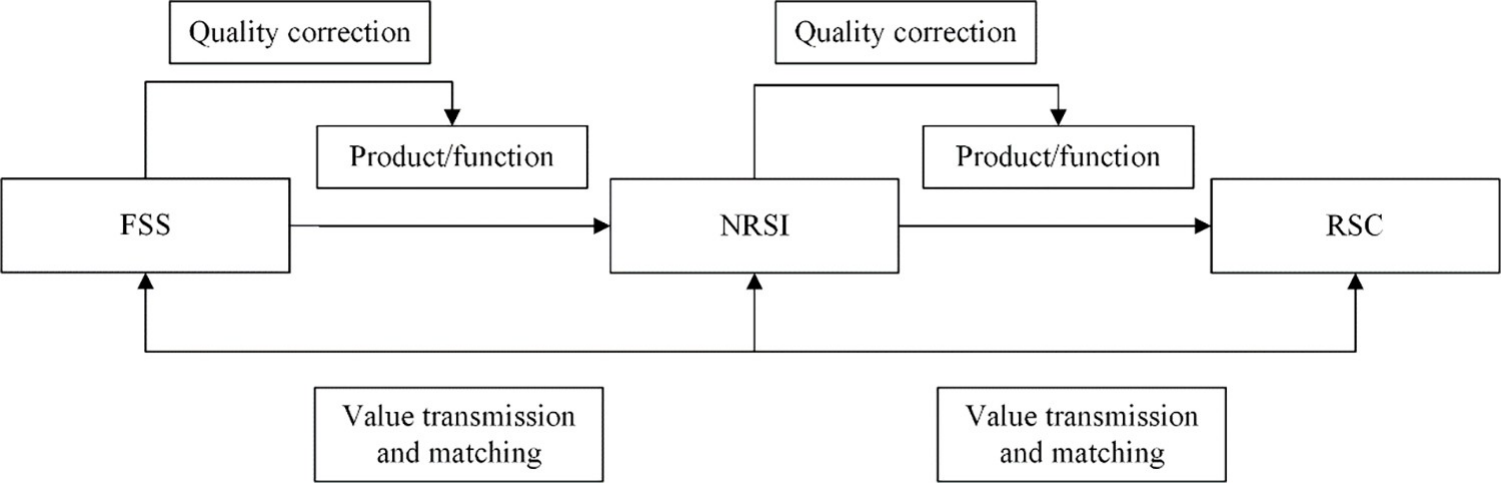
Conceptual diagram of the model.

The conceptual diagram of the new RSSC model is shown in Fig 3. New RSSC refers to the service supply chain strip that to satisfy the service demand of end consumers, taking the new retail enterprise as the core enterprise, integrating the functional services and tangible products of the front-end service providers, and distributing or delivering the service packages including the products, functional services and related services to the end consumers by the new retail service integrators (NRSIs). The new RSSC mainly consists of functional service suppliers (FSSs), NRSIs and retail service consumers. FSSs are the upstream enterprises of the supply chain, which are the providers of tangible products and functional services, as well as the guarantors of product and service quality. The NRSI is the core enterprise of the new RSSC, which needs to take over the FSSs and make secondary quality corrections to the tangible products and functional services, and deliver the products and services that satisfy the quality demand of consumers to the terminal. It is the hub connecting the FSSs and consumers. Consumers are the end-users of tangible products and functional services, and will generate positive or negative quality feedback based on their experience. The new RSSC composed of FSSs, NRSIs and retail service consumers is a typical closed-loop supply chain, where there is a close interaction of value flows among the three to form a more stable synergistic partnership, maximize synergistic effects, and ultimately maximize the benefits of the new RSSC.

The quality improvement of new RSSC is a typical dynamic evolutionary system with multi-body synergy[52]. For the service supply chain system, the complete coordination of the quality improvement behaviors of each participating subject are key to its stable operation. However, in the service supply chain system, the quality improvement behaviors are often uncoordinated due to the fact that multiple subjects do not agree to cooperate, which leads to the failure of the new RSSC quality improvement to reach the optimal steady state, and the subjective emotions of the participating subjects are the important factors affecting the optimal steady state of the quality improvement. Therefore, the quality improvement behaviors of FSSs and NRSIs are analyzed from the perspective of heterogeneous emotions of decision makers and based on the full consideration of consumer quality feedback.

### 3.4. Model construction

**Fig 4:**
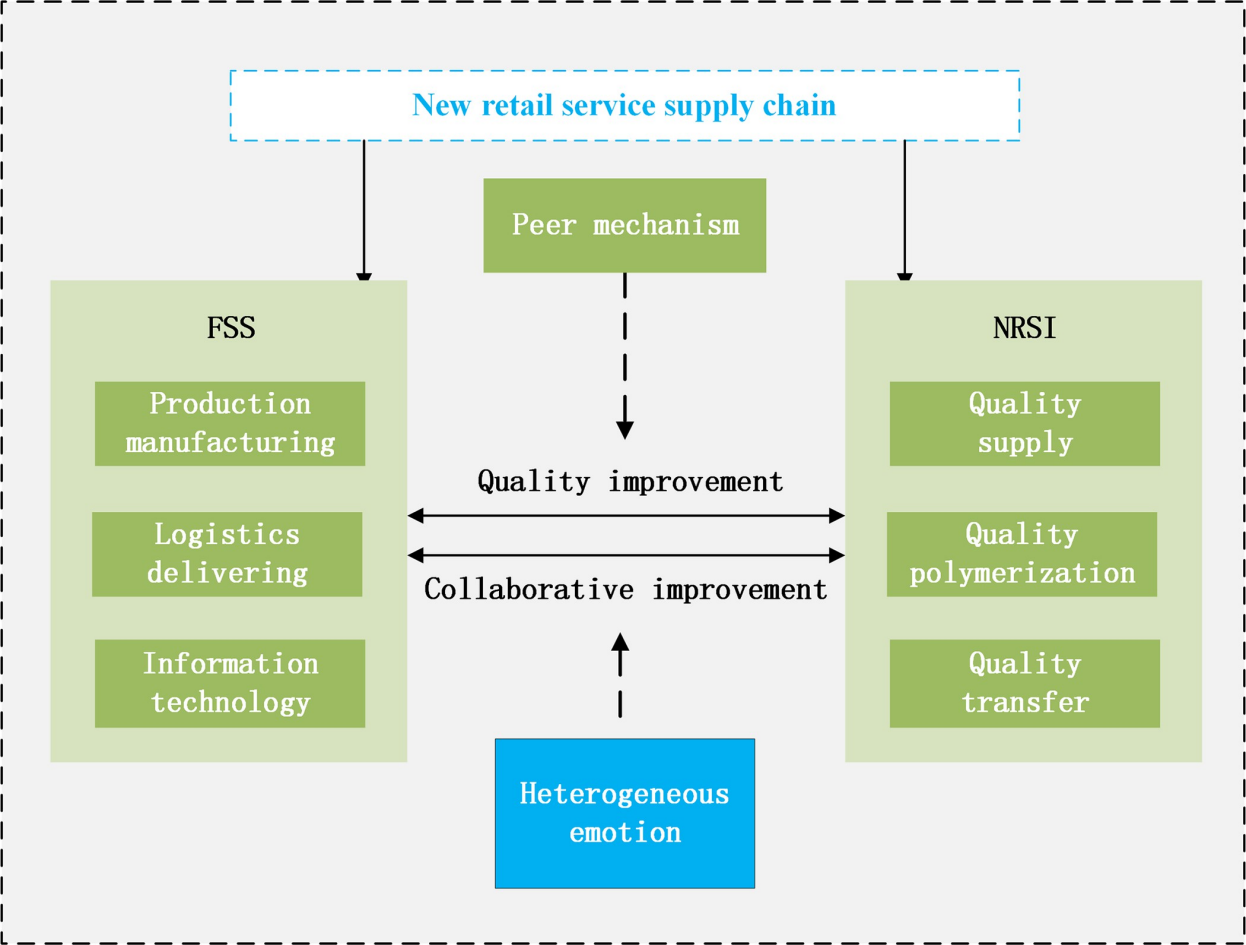
Schematic representation of the evolutionary game model.

The schematic diagram of the new RSSC quality collaborative improvement evolutionary game model is shown in Fig 4.

The specific parameter settings are listed in Table 1.

**Table 1:** Parameters and their meanings.

Parameter	Meaning
*x*	Probability of positive quality improvement for FSSs
*y*	Probability of positive collaborative improvement by NRSIs
*C*_*x*_	Cost of positive quality improvement for FSSs
*C*_*y*_	Cost of positive collaborative improvement for NRSIs
*L*_*a*_	FSSs’ base benefits
*L*_*b*_	NRSIs’ base benefits
*K*_*a*_	FSSs’ quality preferences
*K*_*b*_	NRSIs’ quality preferences
*H*_*a*_	Marginal benefits of quality improvement
*H*_*b*_	“Free-rider” benefits due to spillover effects
*D*	Peer incentives
*F*	Peer punishment
*M*	Positive consumer feedback
*N*	Negative consumer feedback
*L*	Quality risk
*ψ*	Risk factor
*φ*	Risk transfer coefficient
*q*	Probability of risk occurrence
*r*_1_	Emotional intensity of FSSs
*r*_2_	Emotional intensity of NRSIs

The collaborative quality improvement of a new RSSC mainly involves two game subjects, namely: FSSs and NRSIs. Each game subject has certain emotional preferences, and the emotional preferences of each game subject will affect the determination of behavioral decisions. We denote the sentiment intensity of FSSs and NRSIs with *r*_1_ and *r*_2_, respectively.

According to RDEU theory, under the influence of emotion, the subjective probability function becomes *ω*(*p*) = *p*^r^, and *p*(0≤*p*≤1) represents the objective probability of the occurrence of a given decision. When *r*_*i*_ = 1, the subjective probability value is the same as the objective probability value, the game subject does not have emotion and is in rational state; when *r*_*i*_<1, the subjective probability value is higher than the objective probability value, the game subject overestimates the choice probability and shows optimism; when *r*_*i*_>1, the subjective probability value is lower than the objective probability value, the game subject underestimates the probability and is pessimistic.

The strategic choices of FSSs can be categorized into (positive quality improvement, negative quality improvement). NRSIs are motivated to participate in collaborative quality improvement in the context of downstream supply chain firms assuming responsibility for products caused by quality defects of upstream firms. The strategic choices of NRSIs can be categorized as (positive collaborative improvement, negative collaborative improvement). Each player continuously adjusts their strategy in the game process until the strategic evolution reaches an equilibrium state.

The production and sale of the new retail product can generate base benefits, denoted by *L*_*a*_ and *L*_*b*_ for the FSSs and the NRSIs, respectively. Positive quality improvement by the FSSs incurs a certain cost *C*_*x*_, positive collaborative improvement by the service integrator incurs a certain cost *C*_*y*_, *K*_*a*_, and *K*_*b*_ denote the quality preference of the FSSs and the NRSIs, respectively, and *H*_*a*_ denotes the marginal benefit of quality improvement. For the sake of calculation, it is assumed that no cost is incurred by the FSSs and the NRSIs when they adopt a negative strategy. In the process of collaborative quality improvement, there is a quality spillover effect, which allows some node enterprises to “hitchhike”, and the “hitchhiking” benefit due to the quality spillover effect is denoted by *H*_*b*_. The better to incentivize the FSSs and NRSIs to participate actively in collaborative quality improvement, all the node enterprises in the service supply chain contribute to the formation of a peer incentive fund pool to incentivize the FSSs and NRSIs that actively improve quality, with an incentive amount of *D*. Meanwhile, a certain peer penalty is given to the FSSs and NRSIs that are negative about quality improvement, with a penalty of *F*. *D* and *F* are fixed values.

Consumers, as end-users of the new RSSC, are also affected by the quality decisions of FSSs and NRSIs. When FSSs and NRSIs make positive quality improvements, consumers will have a better consumption experience due to the positive “spillover” of quality, which will generate positive feedback to FSSs and NRSIs, and the positive feedback utility of consumers is denoted by *M*. Conversely, quality risks will occur with a probability of *q*. The risk factor is denoted by *ψ*.

According to the principle of risk sharing, when one party chooses positive behavior and the other party chooses negative behavior, the quality risk *L* will be transferred from one party to the other party based on the risk transfer coefficient *φ*. In this case, the consumer will have a poorer experience due to the negative “spillover” of quality, which will generate negative feedback to the FSSs and the NRSIs, and the positive feedback utility of the consumer is denoted by *N*.

Based on the above discussion, the new RSSC quality collaborative improvement evolutionary game payment matrix is shown in Table 2.

**Table 2:** Revenue matrix.

Participating subjects		NRSIs	
		Positive collaborative improvement *y*	Negative collaborative improvement 1−*y*
FSSs	Positive quality improvement	$L_{a}-C_{x}+K_{a}H_{a}+D+M+K_{b}H_{b}$	$L_{a}-C_{x}+K_{a}H_{a}+D+M-\psi Lq$
	*x*	$L_{b}-C_{y}+K_{b}H_{a}+D+M+K_{a}H_{b}$	$L_{b}-F-N+K_{a}H_{b}-\phi \psi Lq$
	Negative quality improvement	$L_{a}+K_{b}H_{b}-F-N-\phi \psi Lq$	$L_{a}-F-N-Lq$
	1−*x*	$L_{b}-C_{y}+K_{b}H_{a}+D+M-\psi Lq$	$L_{b}-F-N-Lq$

## 4. Game analysis

Based on the aforementioned modelling assumptions and RDEU theory, the hierarchical dependent expected utility models of the FSSs and the NRSIs are constructed when adopting different strategies.

### 4.1. Stability analysis of FSSs’ strategies

Based on the evolutionary game replication dynamic equation analysis method based on RDEU theory, for the FSSs, the utilities corresponding to its four strategy choices are ranked according to the relationship between the parameters of cost, benefit, reward, and punishment:

$$\begin{array}{l} L_{a}-C_{x}+K_{a}H_{a}+D+M+K_{b}H_{b}>L_{a}-C_{x}+K_{a}H_{a}+D+M-\psi Lq\\ >L_{a}+K_{b}H_{b}-F-N-\varphi \psi Lq>L_{a}-F-N-Lq \end{array}$$

This leads to the utility, probability, rank and decision weights corresponding to each strategy of the FSSs, as shown in Table 3.

**Table 3:** FSSs’ utility considering emotions.

FSSs’ utility	Probability	Rank	Decision weight
$L_{a}-C_{x}+K_{a}H_{a}+D+M+K_{b}H_{b}$	*xy*	1	*ω*_*A*_(*xy*)
$L_{a}-C_{x}+K_{a}H_{a}+D+M-\psi Lq$	*x*(1−*y*)	1−*xy*	*ω*_*A*_(*x*)−*ω*_*A*_(*xy*)
$L_{a}+K_{b}H_{b}-F-N-\varphi \psi Lq$	(1−*x*)*y*	1−*x*	*ω*_*A*_(*x+y*−*xy*)−*ω*_*A*_(*x*)
$L_{a}-F-N-Lq$	(1−*x*)(1−*y*)	1−*x*−*y*+*xy*	1−*ω*_*A*_(*x+y*−*xy*)

The expected benefits of FSSs choosing “positive quality improvement” is:

$$\begin{array}{l} U_{1x}=[L_{a}-C_{x}+K_{a}H_{a}+D+M+K_{b}H_{b}]y^{r2}+[L_{a}-C_{x}+K_{a}H_{a}+D+M-\psi Lq](1-y^{r2})\\ =L_{a}-C_{x}+K_{a}H_{a}+D+M-(1-y^{r2})\psi Lq+K_{b}H_{b}y^{r2} \end{array}$$ (1)

The expected returns for FSSs choosing “negative quality improvement” can be obtained:

$$\begin{array}{l} U_{2x}=(L_{a}+K_{b}H_{b}-F-N-\varphi \psi Lq)y^{r2}+(L_{a}-F-N-Lq)(1-y^{r2})\\ =L_{a}-F-N+K_{b}H_{b}y^{r2}-(1+\varphi \psi y^{r2}-y^{r2})Lq \end{array}$$ (2)

The average expected return of the FSSs’ strategy choice is:

$$\begin{array}{l} \bar{U_{x}}=(L_{a}-C_{x}+K_{a}H_{a}+D+M+K_{b}H_{b})\omega_{A}(xy)+(L_{a}-C_{x}+K_{a}H_{a}+D+M-\psi Lq)[\omega_{A}(x)-\omega_{A}(xy)]\\ +(L_{a}+K_{b}H_{b}-F-N-\varphi \psi Lq)[\omega_{A}(x+y-xy)-\omega_{A}(x)]+(L_{a}-F-N-Lq)[1-\omega_{A}(x+y-xy)]\\ =(K_{b}H_{b}+\psi Lq){\left(xy\right)}^{r1}+(-C_{x}+K_{a}H_{a}+D+M-\psi Lq-K_{b}H_{b}+F+N+\varphi \psi Lq)x^{r1}\\ +(K_{b}H_{b}-\varphi \psi Lq+Lq){\left(x+y-xy\right)}^{r1}+L_{a}-F-N-Lq \end{array}$$ (3)

If the expected utility of a certain strategy is larger than the group’s average expected value, the percentage of individuals who adopt that strategy will increase. As a result, the dynamic equation of re plication can express the mechanism of dynamic strategy adjustment by game parties and then evaluate the strategy selection law of decision makers. The dynamic equation of government regulator replication is calculated using the asymmetric replication dynamic evolution approach. The replication dynamic equation of FSSs is:

$$\begin{array}{l} F(x)=dx/dt=x^{r1}(U_{1x}-\bar{U_{x}})=x^{r1}[F+N+Lq-C_{x}+K_{a}H_{a}+D+M\\ -(1-y^{r2})\psi Lq+K_{b}H_{b}y^{r2}-(K_{b}H_{b}+\psi Lq){\left(xy\right)}^{r1}-(-C_{x}+K_{a}H_{a}+D+\\ M-\psi Lq-K_{b}H_{b}+F+N+\varphi \psi Lq)x^{r1}-(K_{b}H_{b}-\varphi \psi Lq+Lq){\left(x+y-xy\right)}^{r1}] \end{array}$$ (4)

From Eq (4), it can be obtained that the FSSs’ choice of active quality improvement strategy can achieve local stabilization when *x* = 0, *x* = 1, or *x* = x*.

### 4.2. Strategy stability analysis for NRSIs

For the NRSIs, the utility ranking corresponding to its four strategy choices is based on the relationship between the parameters of cost, benefit, reward, and penalty:

$$\begin{array}{l} L_{b}-C_{y}+K_{b}H_{a}+D+M+K_{a}H_{b}>L_{b}-C_{y}+K_{b}H_{a}+D+M-\psi Lq\\ >L_{b}-F-N+K_{a}H_{b}-\varphi \psi Lq>L_{b}-F-N-Lq \end{array}$$

This leads to the utility, probability, rank and decision weights corresponding to each strategy of the NRSIs, as shown in Table 4.

**Table 4:** NRSIs’ utility considering emotions.

NRSIs’ utility	Probability	Rank	Decision weight
$L_{b}-C_{y}+K_{b}H_{a}+D+M+K_{a}H_{b}$	*xy*	1	*ω*_*B*_(*xy*)
$L_{b}-C_{y}+K_{b}H_{a}+D+M-\psi Lq$	(1−*x*)*y*	1−*xy*	*ω*_*B*_(*y*)−*ω*_*B*_(x*y*)
$L_{b}-F-N+K_{a}H_{b}-\varphi \psi Lq$	*x*(1−*y*)	1−*y*	*ω*_*B*_(*x*+*y*−*xy*)−*ω*_*B*_(*y*)
$L_{b}-F-N-Lq$	(1−*x*)(1−*y*)	1−*x*−*y*+*xy*	1−*ω*_*B*_(*x+y*−*xy*)

The expected benefit to the NRSIs of choosing “Active collaborative improvement” is:

$$\begin{array}{l} U_{1y}=[L_{b}-C_{y}+K_{b}H_{a}+D+M+K_{a}H_{b}]x^{r1}+[L_{b}-C_{y}+K_{b}H_{a}+D+M-\psi Lq](1-x^{r1})\\ =L_{b}-C_{y}+K_{b}H_{a}+D+M-(1-x^{r1})\psi Lq+K_{a}H_{b}x^{r1} \end{array}$$ (5)

The expected return for a NRSIs choosing “Negative collaborative improvement” is:

$$\begin{array}{l} U_{2y}=(L_{b}-F-N+K_{a}H_{b}-\varphi \psi Lq)x^{r1}+(L_{b}-F-N-Lq)(1-x^{r1})\\ =L_{b}-F-N+K_{a}H_{b}-(1+\varphi \psi x^{r1}-x^{r1})Lq \end{array}$$ (6)

The average expected return of the FSSs’ strategy choice is:

$$\begin{array}{l} \bar{U_{y}}=(L_{b}-C_{y}+K_{b}H_{a}+D+M+K_{a}H_{b})\omega_{B}(xy)+(L_{b}-C_{y}+K_{b}H_{a}+D+M-\psi Lq)[\omega_{B}(y)-\omega_{B}(xy)]\\ +(L_{b}-F-N+K_{a}H_{b}+\varphi \psi Lq)[\omega_{B}(x+y-xy)-\omega_{B}(y)]+(L_{b}-F-N-Lq)[1-\omega_{B}(x+y-xy)]\\ =(K_{a}H_{b}+\psi Lq){\left(xy\right)}^{r2}+(-C_{y}+K_{b}H_{a}+D+M-\psi Lq+F+N-K_{a}H_{b}+\varphi \psi Lq){\left(y\right)}^{r2}\\ +(K_{a}H_{b}-\varphi \psi Lq+Lq){\left(x+y-xy\right)}^{r2}+L_{b}-F-N-Lq \end{array}$$ (7)

The replication dynamic equation for NRSIs is:

$$\begin{array}{l} F(y)=dy/dt=y^{r2}(U_{1y}-\bar{U_{y}})=y^{r2}[F+N+Lq-C_{y}+K_{b}H_{a}+D+M\\ -(1-x^{r1})\psi Lq+K_{a}H_{b}x^{r1}-(K_{a}H_{b}+\psi Lq){\left(xy\right)}^{r2}-(-C_{y}+K_{b}H_{a}+D+M\\ -\psi Lq+F+N-K_{a}H_{b}+\varphi \psi Lq){\left(y\right)}^{r2}-(K_{a}H_{b}-\varphi \psi Lq+Lq){\left(x+y-xy\right)}^{r2}] \end{array}$$ (8)

From Eq (8), the NRSIs can achieve local stabilization by choosing an active collaborative improvement strategy when *y* = 0, *y* = 1 or *y* = y*.

### 4.3. Strategy portfolio stability analysis

From the analysis in Sections 3.1 and 3.2, the five local equilibrium points of the evolutionary game model are *E*_1_(0,0), *E*_2_(0,1), *E*_3_(1,0), *E*_4_(1,1)and *E*_5_(x*,y*), respectively.

The strategic choices of game subjects are not only affected by their own utility, but also by the influences of stakeholders. Therefore, it is more realistic to further estimate the stability of strategy portfolio on the basis of evaluating the stability of individual subject’s strategy. According to the stability analysis of the evolutionary game, the stability of the strategy portfolio of each game subject can be judged according to the Lyapunov indirect method, and the Jacobian matrix of the game model is obtained from Eqs (4) and (8):

$$J=[\begin{array}{l} \partial F(x)/\partial x\;\partial F(x)/\partial y\\ \partial F(y)/\partial x\;\partial F(y)/\partial y \end{array}]$$ (9)

Among them:

$$\begin{array}{l} \partial F(x)/\partial x=r1x^{r1-1}[F+N+Lq-C_{x}+K_{a}H_{a}+D+M-(1-y^{r2})\psi Lq+K_{b}H_{b}y^{r2}-(K_{b}H_{b}+\psi Lq){\left(xy\right)}^{r1}\\ -(-C_{x}+K_{a}H_{a}+D+M-\psi Lq-K_{b}H_{b}+F+N+\varphi \psi Lq)x^{r1}-(K_{b}H_{b}-\varphi \psi Lq+Lq){\left(x+y-xy\right)}^{r1}]\\ +x^{r1}[-(K_{b}H_{b}+\psi Lq)r1x^{r1-1}y^{r1}-(-C_{x}+K_{a}H_{a}+D+M-\psi Lq-K_{b}H_{b}+F+N+\varphi \psi Lq)r1x^{r1-1}-\\ \left(K_{b}H_{b}-\varphi \psi Lq+Lq)r1(1-y){\left(x+y-xy\right)}^{r1-1}\right] \end{array}$$ (10)

$$\begin{array}{l} \partial F(y)/\partial y=r2y^{r2-1}[F+N+Lq-C_{y}+K_{b}H_{a}+D+M-(1-x^{r1})\psi Lq+K_{a}H_{b}x^{r1}-(K_{a}H_{b}+\psi Lq){\left(xy\right)}^{r2}\\ -(-C_{y}+K_{b}H_{a}+D+M-\psi Lq+F+N-K_{a}H_{b}+\varphi \psi Lq){\left(y\right)}^{r2}-(K_{a}H_{b}-\varphi \psi Lq+Lq){\left(x+y-xy\right)}^{r2}]\\ +y^{r2}[-(K_{a}H_{b}+\psi Lq)x^{r2}r2y^{r2-1}-(-C_{y}+K_{b}H_{a}+D+M-\psi Lq+F+N-K_{a}H_{b}+\varphi \psi Lq)r2y^{r2-1}\\ -(K_{a}H_{b}-\varphi \psi Lq+Lq)r2(1-x){\left(x+y-xy\right)}^{r2-1}] \end{array}$$ (11)

$$\begin{array}{l} \partial F(x)/\partial y=x^{r1}[r2y^{r2-1}\psi Lq+r2K_{b}H_{b}y^{r2-1}-r1(K_{b}H_{b}+\psi Lq)x^{r1}y^{r1-1}\\ -r1(K_{b}H_{b}-\varphi \psi Lq+Lq)(1-x){\left(x+y-xy\right)}^{r1-1}] \end{array}$$ (12)

$$\begin{array}{l} \partial F(y)/\partial x=y^{r2}[r1x^{r1-1}\psi Lq+r1K_{a}H_{b}x^{r1-1}-(K_{a}H_{b}+\psi Lq)r2x^{r2-1}y^{r2}\\ -(K_{a}H_{b}-\varphi \psi Lq+Lq)r2(1-y){\left(x+y-xy\right)}^{r2-1}] \end{array}$$ (13)

Det(J)=(∂F(x)/∂x)(∂F(y)/∂y)−(∂F(x)/∂y)(∂F(y)/∂x) (14)

Tr(J)=∂F(x)/∂x+∂F(y)/∂y (15)

Since the value of Jacobian matrix is related to the value of model variables, the value of the Jacobian matrix is different under different emotional states of the game subjects, and the equilibrium points obtained also differ. Therefore, this paper evaluates the stability of the strategy combinations of FSSs and NRSIs under four scenarios: (rational, rational), (emotional, emotional), (rational, emotional), and (emotional, rational) based on the different emotional states of the game subjects.

#### 4.3.1. Scenario 1: Rational FSSs, rational NRSIs

When the FSSs are rational and the NRSIs are rational, at this time the sentiment parameter *r*_1_ = 1,*r*_2_ = 1. Substituting the sentiment parameter into each replicated dynamic equation, at this time the strategy portfolio stability analysis is shown in Table 5.

**Table 5:** Stability analysis of strategy portfolios under the scenario 1.

Equilibrium point	*Det*(*J*)	*Tr*(*J*)	Stability
*E*_1_(0,0)	×	×	Saddle point
*E*_2_(1,0)	×	×	Saddle point
*E*_3_(0,1)	×	×	Saddle point
*E*_4_(1,1)	×	×	Saddle point
*E*_5_(x*,y*)	Stability depends on specific values		

From Table 5, when the FSSs are rational and the NRSIs are rational, there are four saddle points in the system, which are *E*_1_(0,0), *E*_2_(1,0), *E*_3_(0,1) and *E*_4_(1,1). The stability of the local equilibrium point *E*_5_(p*,e*) cannot be determined, and its stability depends on the specific values.

#### 4.3.2. Scenario 2: Emotional FSSs, emotional NRSIs

When the FSSs are emotional and the NRSIs are emotional, at this time the sentiment parameters, are such that *r*_1_≠1,*r*_2_≠1, the sentiment parameters are brought into each replicated dynamic equation, at this time the strategy portfolio stability analysis is displayed in Table 6.

**Table 6:** Stability analysis of strategy portfolio under the scenario 2.

Equilibrium point	*Det*(*J*)	*Tr*(*J*)	Stability
*E*_1_(0,0)	0	0	Unstable
*E*_2_(1,0)	0	×	Unstable
*E*_3_(0,1)	0	×	Unstable
*E*_4_(1,1)	×	×	Saddle point
*E*_5_(x*,y*)	Stability depends on specific values and emotional intensity		

According to Table 6, when the FSSs are emotional and the NRSIs are emotional, the system has three instability points *E*_1_(0,0), *E*_2_(1,0), *E*_3_(0,1), a saddle point *E*_4_(1,1), and at the same time, because the emotional intensity of the FSSs and the NRSIs is unknown, *i*.*e*., the specific value of the emotional parameter *r*_1_、*r*_2_ cannot be determined, the stability of the local equilibrium point *E*_5_(p*,e*) cannot be determined, and its stability is dependent on the specific value and the intensity of the emotion.

#### 4.3.3. Scenario 3: Rational FSSs, emotional NRSIs

When the FSSs are rational and the NRSIs are emotional, then the emotional parameters *r*_1_ = 1,*r*_2_≠1. The emotional parameters are brought into the dynamic equations of each replication, at this time, the stability analysis of the strategy portfolio is shown in Table 7.

**Table 7:** Stability analysis of strategy portfolios under the scenario 3.

Equilibrium point	*Det*(*J*)	*Tr*(*J*)	Stability
*E*_1_(0,0)	0	×	unstable
*E*_2_(1,0)	0	×	unstable
*E*_3_(0,1)	×	×	saddle point
*E*_4_(1,1)	×	×	saddle point
*E*_5_(x*,y*)	Stability depends on specific values and emotional intensity		

As can be seen from Table 7, when the FSSs are rational and the NRSIs are emotional, the system exists two instability points *E*_1_(0,0), *E*_2_(1,0), two saddle points *E*_3_(0,1), *E*_4_(1,1), at the same time, the stability of the local equilibrium point cannot be determined due to the NRSIs’; emotional intensity is unknown, *i*.*e*., the specific value of the emotional parameter *r*_2_ cannot be determined, and the stability of the local equilibrium point *E*_5_(p*,e*) is not determined, and the stability of the local equilibrium point is dependent on the specific value and the intensity of the emotional intensity.

#### 4.3.4. Scenario 4: Emotional FSSs, rational NRSIs

When the FSSs are emotional and the NRSIs are rational, the emotional parameters *r*_1_≠1,*r*_2_ = 1. The emotional parameters are brought into the dynamic equations of each replication, and at this time the stability analysis of the strategy portfolio is shown in Table 8.

**Table 8:** Stability analysis of strategy portfolios under the scenario 4.

Equilibrium point	*Det*(*J*)	*Tr*(*J*)	Stability
*E*_1_(0,0)	0	×	Unstable
*E*_2_(1,0)	×	×	Saddle point
*E*_3_(0,1)	0	×	Unstable
*E*_4_(1,1)	×	×	Saddle point
*E*_5_(x*,y*)	Stability depends on specific values and emotional intensity		

As shown in Table 8, when the FSSs are emotional and the NRSIs are rational, the system has two points of instability *E*_1_(0,0) and *E*_3_(0,1), and two saddle points *E*_2_(1,0) and *E*_4_(1,1). Meanwhile, the stability of the local equilibrium point cannot be determined because the emotional intensity of the FSSs is unknown, *i*.*e*., the specific value of the emotional parameter *r*_1_ cannot be determined, and the stability of the local equilibrium point *E*_5_(p*,e*) is also not determined, and the stability of the system is dependent on the specific value and the emotional intensity.

## 5. Simulation analysis

The more intuitively to study the evolutionary patterns between functional service providers and retail service integrators, the effects of key parameters and heterogeneous emotions on evolutionary stability are assessed. In this paper, taking China’s JS retail chain enterprise as an example, MATLAB™ is used to conduct simulation analysis. JS is a comprehensive retail chain enterprise, which began to enter the field of new retail in 2015, constructed an omni-channel retail service system of on-line and off-line interactions, and shifted from traditional wholesaling and retailing to a modern RSSC operation enterprise mainly focusing on the integration of new retail services, and in recent years, the enterprise’s new retail model is remarkable, but there is also the problem of how to coordinate the service quality behaviors of FSS and NRSI as well as the optimal returns of the overall RSSC. After field interviews and surveys of the enterprise, the parameters are set with reference to the literature [35,38,40], and adjusted according to the opinions of experts in related fields; the specific parameter settings are shown in Table 9.

**Table 9:** Parameter settings.

Parameter	*x*	*y*	*C*_*x*_	*C*_*y*_	*L*_*a*_	*L*_*b*_	*K*_*a*_	*K*_*b*_	*H*_*a*_	*H*_*b*_
Initial value	0.4	0.4	4	4	5.5	5	0.4	0.3	2	2
Parameter	*D*	*F*	*M*	*N*	*L*	*ψ*	*ϕ*	*q*	*r*_1_	*r*_2_
Initial value	1	1	0.8	0.5	1	0.2	0.2	0.5	1	1

### 5.1. Influences of key parameters

#### 5.1.1. Effect of quality preference

**Fig 5:**
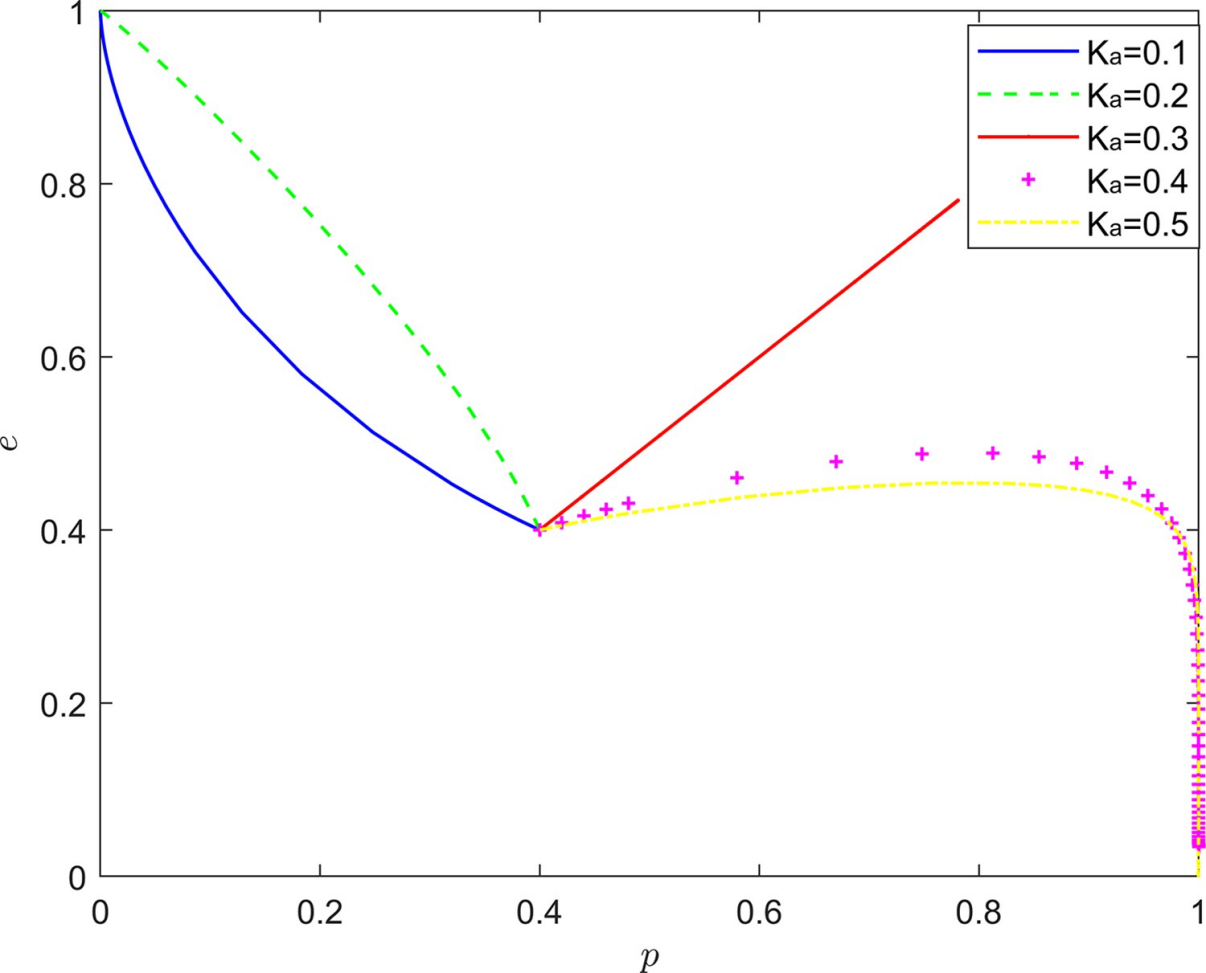
Effect of *K*_*a*_ on system evolution.

Taking {*K*_*a*_ = 0.1, *K*_*a*_ = 0.2, *K*_*a*_ = 0.3, *K*_*a*_ = 0.4, *K*_*a*_ = 0.5}, the strategy evolution process and results are shown in Fig 5.

**Fig 6:**
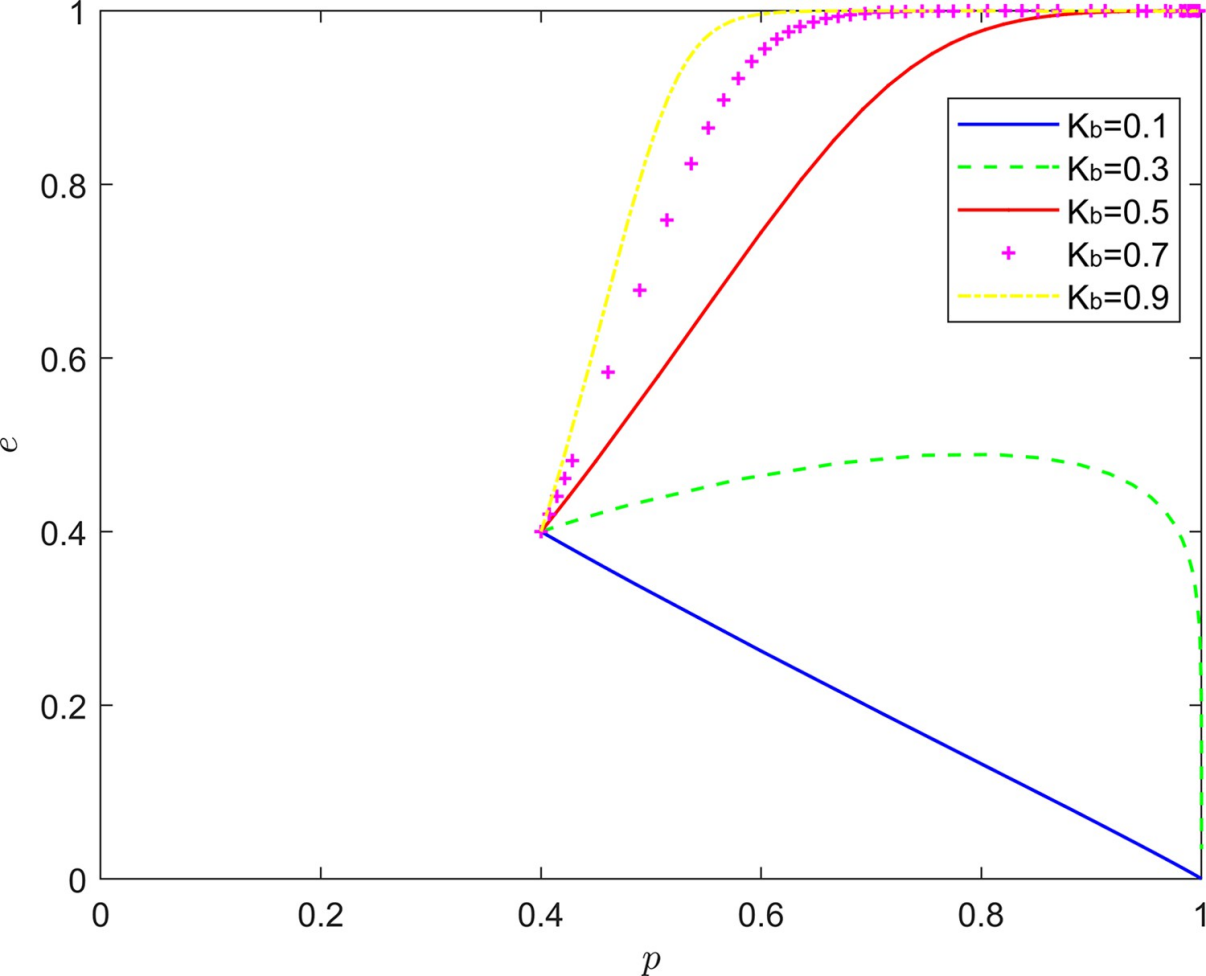
Effect of *K*_*b*_ on system evolution.

Taking {*K*_*b*_ = 0.1, *K*_*a*_ = 0.3, *K*_*a*_ = 0.5, *K*_*a*_ = 0.7, *K*_*a*_ = 0.9}, the strategic evolution process and results are shown in Fig 6.

As shown in Figs 5 and 6, there is a non-linear relationship between quality preferences of FSSs and NRSIs and collaborative quality improvement. When the quality preference of FSSs is too low, the probability of their positive quality inputs will be maintained at a low level; when the quality preference of FSSs is too high, the “free-riding” behavior of NRSIs will be intensified, which will lead to the deterioration of the quality improvement effect of the new RSSC with the increase of the quality preference of FSSs; when the quality preference of NRSIs is too high, the quality improvement effect of the new RSSC will deteriorate. When the quality preference of NRSIs is too low, the probability of their participation in collaborative quality improvement will be maintained at a low level, and when the quality preference of FSSs is too high, collaborative quality improvement will be inelastic, and the collaborative quality improvement effect of the new RSSC will not change according to the change of quality preference. This non-linear characteristic makes the quality preference of FSSs and NRSIs better exert their positive effect on the collaborative quality improvement of the new RSSC only when their quality preference is in the moderate range.

#### 5.1.2. Effect of peer mechanisms

**Fig 7:**
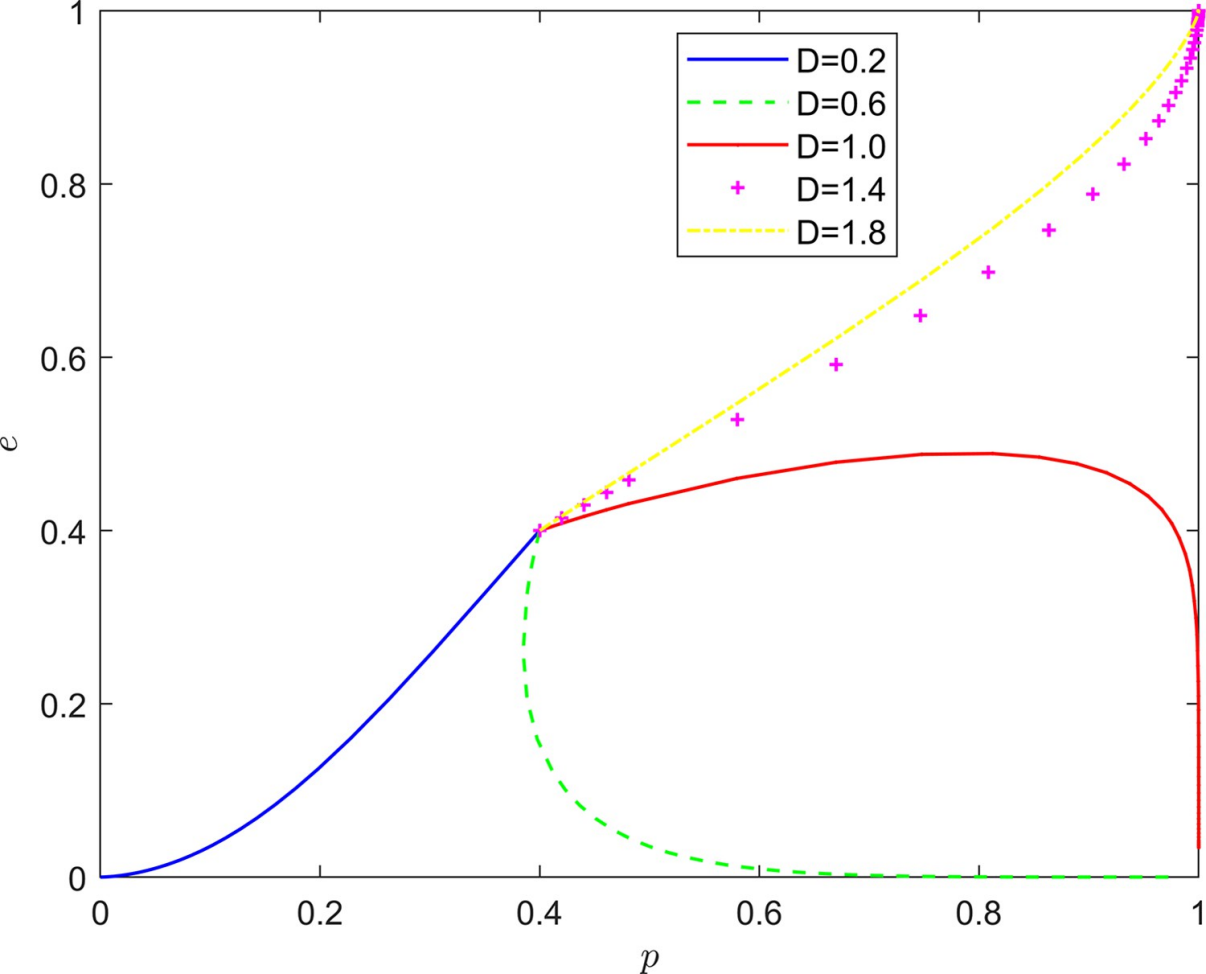
Effect of *D* on system evolution.

Taking {*D* = 0.2, *D* = 0.6, *D* = 1.0, *D* = 1.4, *D* = 1.8}, the strategy evolution process and results are shown in Fig 7.

**Fig 8:**
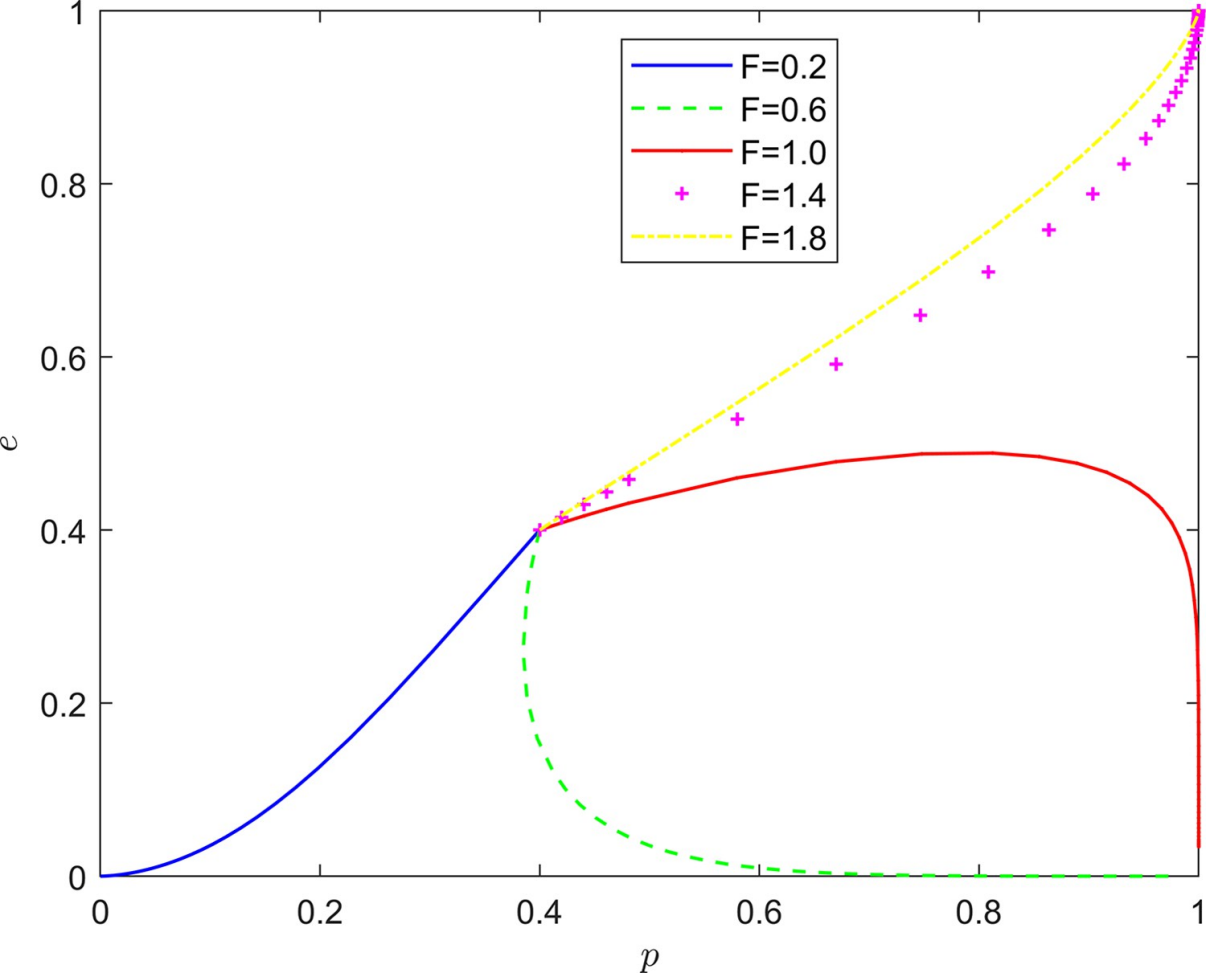
Effect of *F* on system evolution.

Taking {*F* = 0.2, *F* = 0.6, *F* = 1.0, *F* = 1.4, *F* = 1.8}, the strategy evolution process and results are illustrated in Fig 8.

As illustrated in Figs 7 and 8, the peer mechanism can significantly affect the effect of collaborative quality improvement in the new RSSC, and the peer mechanism can change the location of the equilibrium point of system evolution. By observing the horizontal and vertical coordinates of the equilibrium point under different peer mechanisms, it is found that the equilibrium point of the system evolution gradually changes from (0,0) to (1,0) and finally stabilizes at the vicinity of (1,1) as both the incentive and punishment increase in intensity. It can be seen that the introduction of the peer mechanism can significantly increase the probability of active quality improvement by FSSs and active participation in quality improvement by NRSIs, so that the stabilization strategy of the quality improvement system of the new RSSC can evolve in the direction of “Pareto optimality”.

#### 5.1.3. Impact of feedback mechanisms

**Fig 9:**
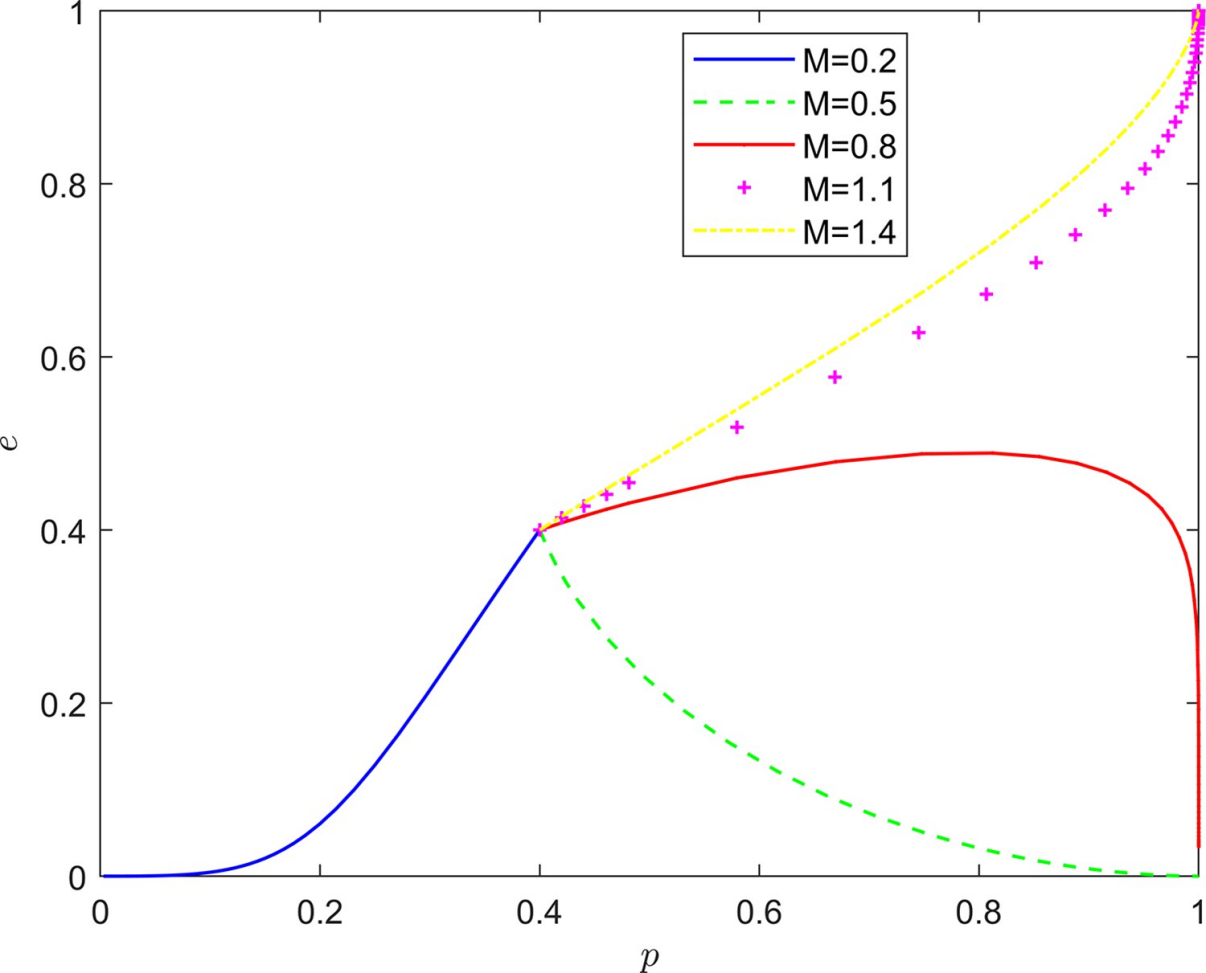
Effect of *M* on system evolution.

Taking {*M* = 0.2, *M* = 0.5, *M* = 0.8, *M* = 1.1, *M* = 1.4}, the strategic evolution and results are shown in Fig 9.

**Fig 10:**
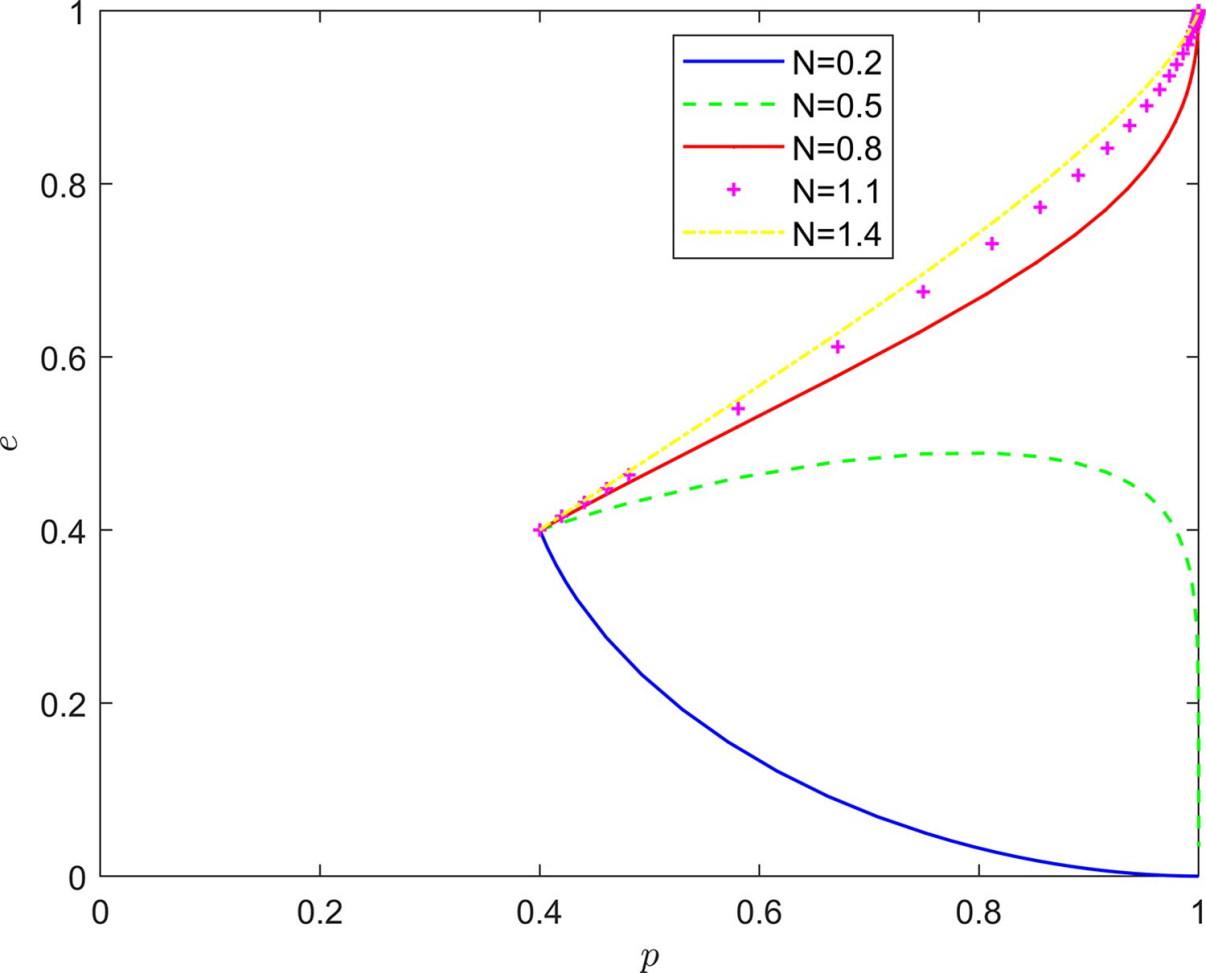
Effect of *N* on system evolution.

Taking {*N* = 0.2, *N* = 0.5, *N* = 0.8, *N* = 1.1, *N* = 1.4}, the strategic evolution and results are shown in Fig 10.

As shown in Figs 9 and 10, the feedback mechanism can significantly affect the effect of collaborative quality improvement in the new RSSC, and the effectiveness of the positive feedback mechanism is better than that of the negative feedback mechanism. By observing the position of the horizontal and vertical coordinates of the equilibrium point under different feedback mechanisms, it can be found that: under the positive feedback mechanism, with the strengthening of the feedback mechanism, the equilibrium point of the system evolution is gradually changed from (0,0) to (1,0) and finally stabilized at the vicinity of (1,1). Under the negative feedback mechanism, the equilibrium point of system evolution gradually changes from (1,0) to (1,1) as the feedback mechanism is strengthened. It can be seen that the introduction of the feedback mechanism can significantly increase the probability of active quality improvement by the FSSs and the active participation of the NRSIs in the collaborative quality improvement, so that the stabilization strategy of the collaborative quality improvement system of the new RSSC can evolve in the direction of “Pareto optimality”.

#### 5.1.4. Impact of risk mechanisms

**Fig 11:**
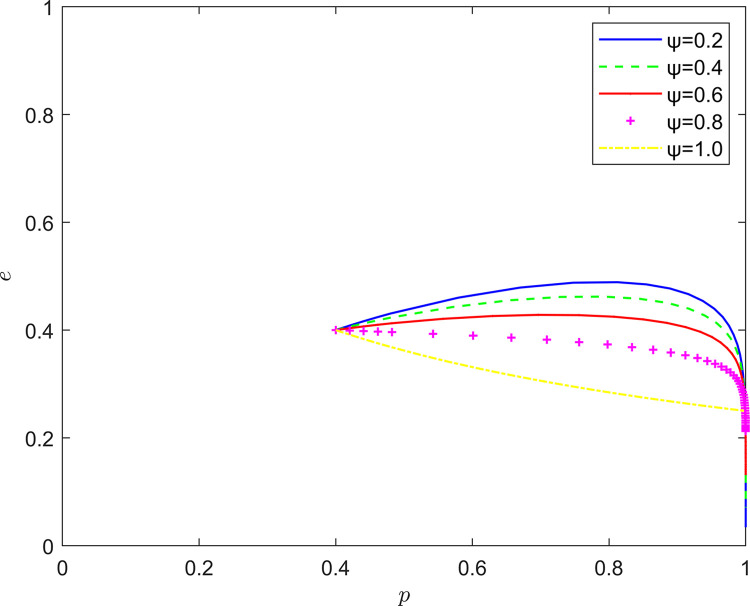
Effect of *ψ* on system evolution.

Taking {*ψ* = 0.2, *ψ* = 0.4, *ψ* = 0.6, *ψ* = 0.8, *ψ* = 1.0}, the strategic evolution and results are shown in Fig 11.

**Fig 12:**
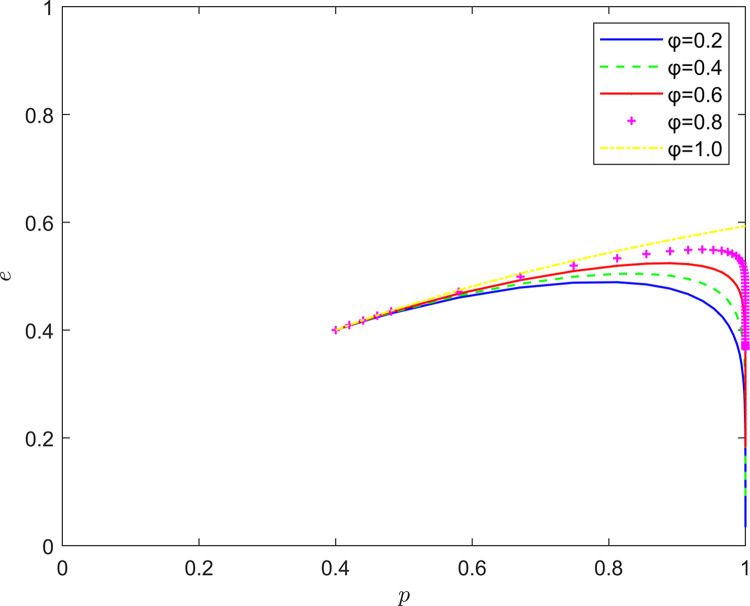
Effect of *φ* on system evolution.

Taking {*φ* = 0.2, *φ* = 0.4, *φ* = 0.6, *φ* = 0.8, *φ* = 1.0}, the strategic evolution and results are shown in Fig 12.

**Fig 13:**
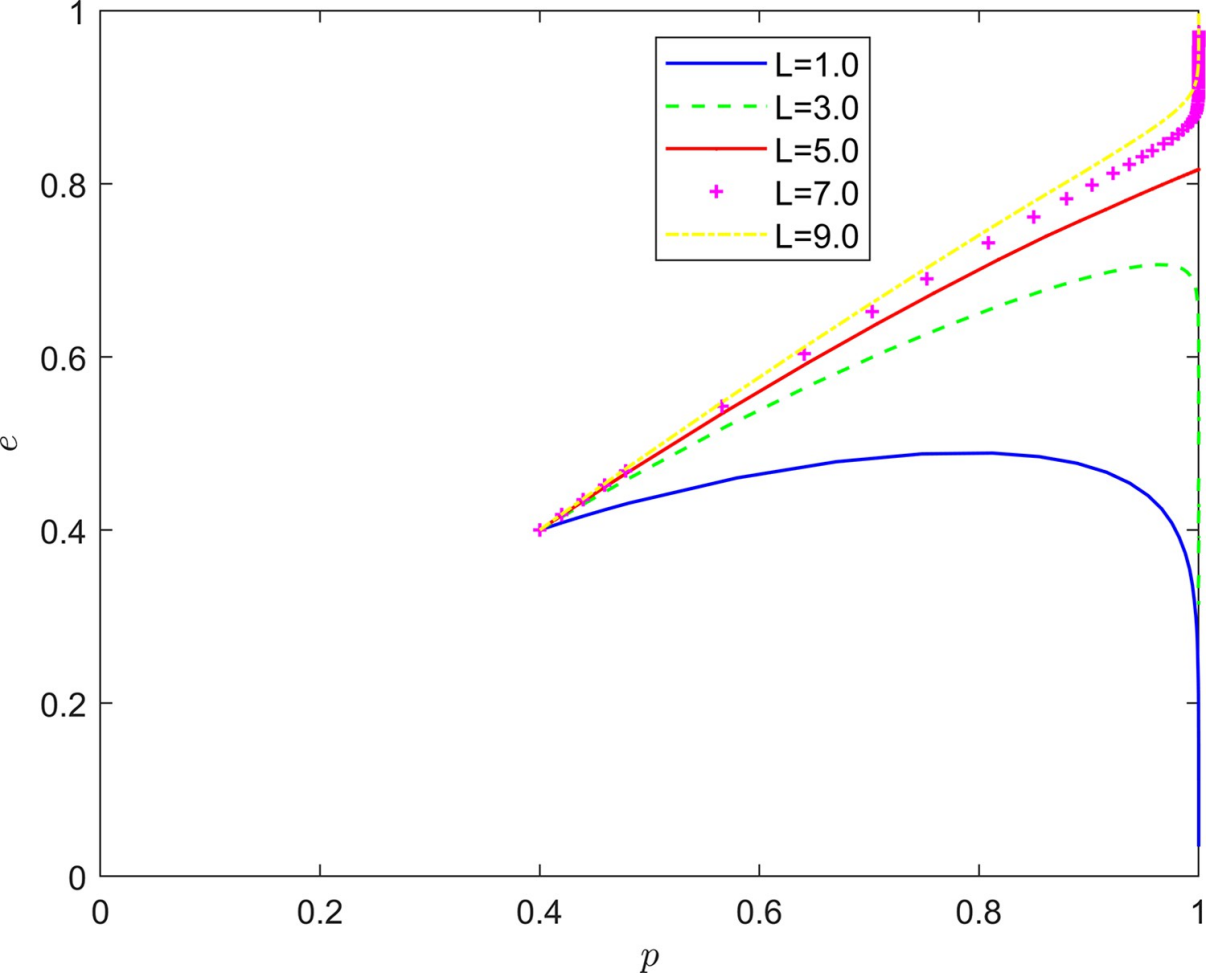
Effect of *L* on system evolution.

Taking {*L* = 1.0, *L* = 3.0, *L* = 5.0, *L* = 7.0, *L* = 9.0}, the strategic evolution and results are shown in Fig 13.

As illustrated in Figs 11–13, the risk mechanism can significantly affect the effect of quality collaborative improvement in the new RSSC, and the effectiveness of the role of different key elements varies. In terms of the risk coefficient, as the risk coefficient increases, the equilibrium point of system evolution moves vertically to a certain extent, and the probability of active participation of NRSIs in quality collaborative improvement is enhanced, and the enhancement of the willingness to participate in quality collaborative improvement of NRSIs is mainly attributed to the enhancement of the probability of the occurrence of quality risk.

In terms of the risk transfer coefficient, as the risk transfer coefficient increases, the probability of NRSIs actively participating in quality improvement is also enhanced, and the enhancement is greater than the probability of enhancement under the influence of the risk coefficient, and the enhancement of the willingness to participate in the quality collaboration of the NRSIs is mainly due to the transfer of the quality risk and the transfer of the quality risk. In terms of risk cost, the equilibrium point of system evolution gradually changes from (1,0) to (1,1) with the increase of quality risk cost, and the increase of NRSIs’ willingness to participate in quality collaboration mainly comes from the increase of potential risk cost.

### 5.2. Effects of heterogeneous emotions

In Section 4.3, the strategy portfolio stability under four scenarios, (rational, rational), (emotional, emotional), (rational, emotional), and (emotional, rational) is assessed, based on the differences in the emotional states of the new retail service providers and the new retail functional integrators. Based on the RDEU theory, the decision maker’s emotional state can be further subdivided into optimism and pessimism. Here, the evolutionary stability of the system under different emotional states will be analyzed further on the basis of the analysis in Section 3.3.

#### 5.2.1. Analysis of (Rational, rational) state

**Fig 14:**
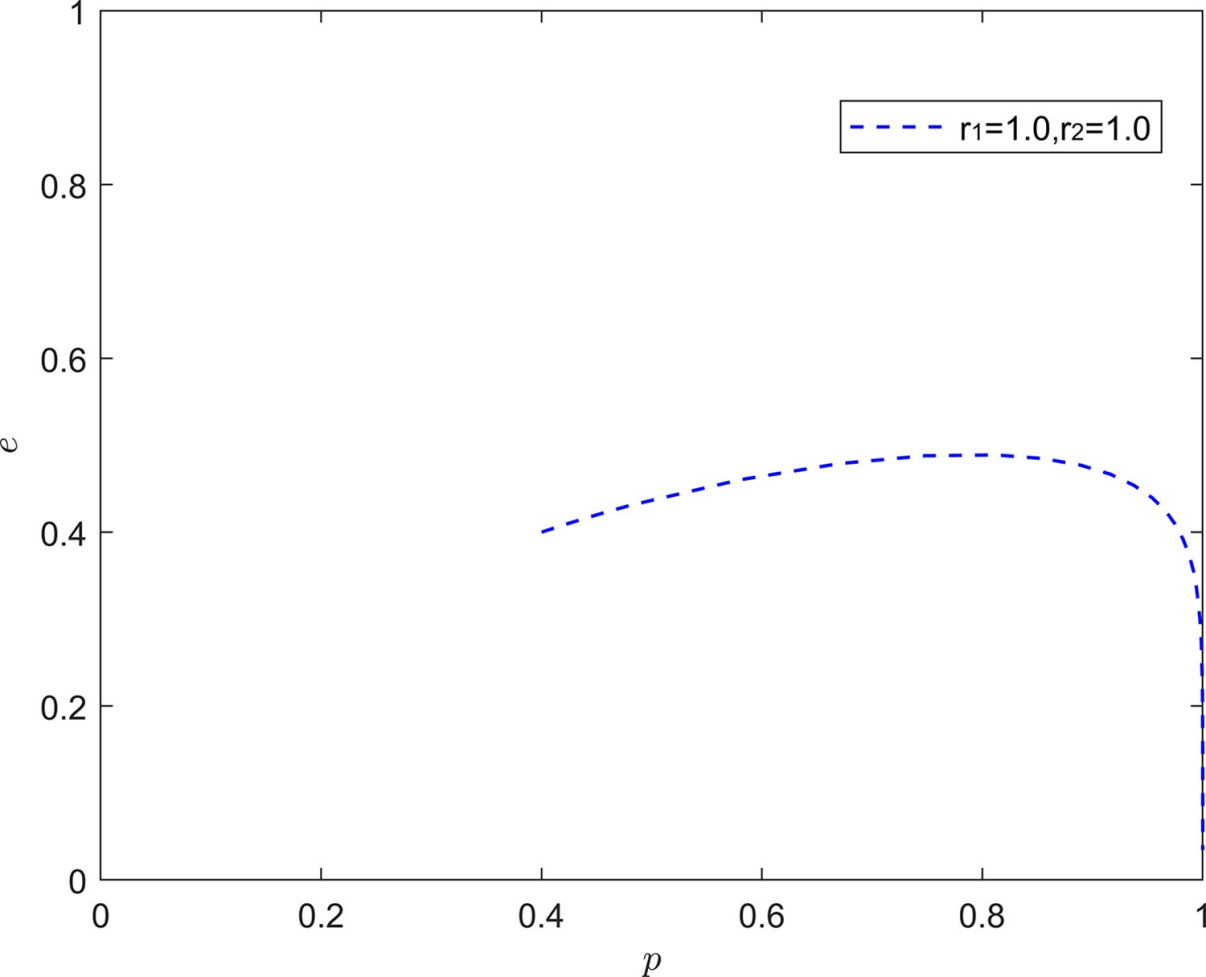
Evolution of game strategies in a (rational, rational) state.

Fig 14 illustrates the equilibrium strategy when the FSSs are rational and the NRSIs are rational. When the system evolutionary stability point is (1,0), *i*.*e*., the FSSs choose positive quality improvement and the NRSIs choose negative collaborative improvement.

This is close to the real situation. The improvement of product quality in the new RSSC is mainly due to the investment in quality improvement by the FSSs, and due to the spillover effect of the quality investment, the NRSIs can attain most of the incremental benefits, and the NRSIs in the downstream of the new RSSC can “ride on the bandwagon” without paying any cost. NRSIs in the downstream of the new RSSC will enjoy the positive externalities of quality investment at no cost to them.

#### 5.2.2. (Optimistic, optimistic) state analysis

**Fig 15:**
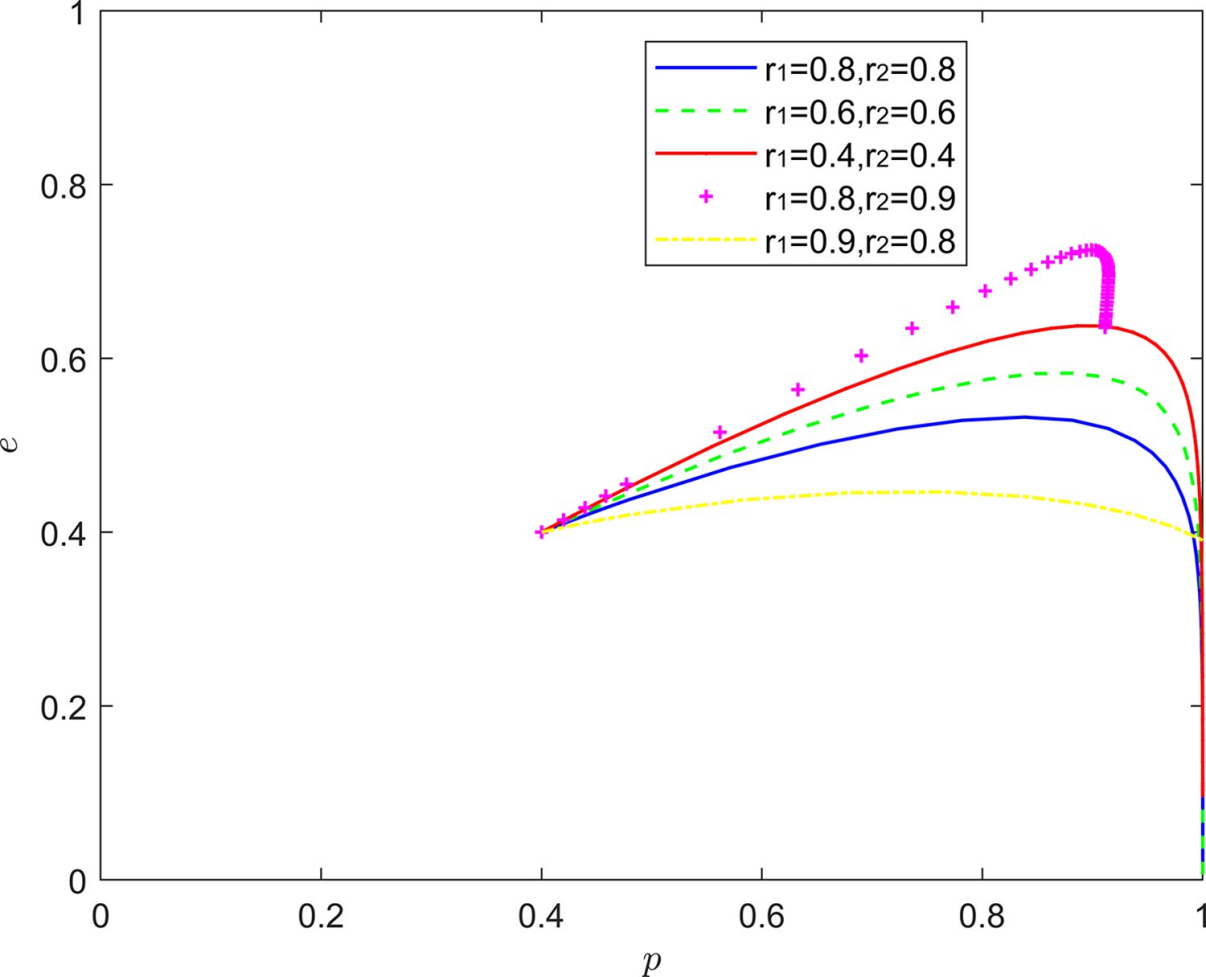
Evolution of game strategies in an (optimistic, optimistic) state.

Fig 15 shows the equilibrium strategy when the FSSs are optimistic and the NRSIs are optimistic (*r*_1_<1,*r*_2_<1). When the optimism of the FSSs and the NRSIs is deepening (*r*_1_ = *r*_2_), the steady state of the system evolution does not change and the convergence rate of the system gradually slows down. When there is a difference in the intensity of optimism between the two, a new mixed strategy Nash equilibrium point emerges in the system evolution. When the optimism of the FSSs is stronger than that of the NRSIs (*r*_1_<*r*_2_), the equilibrium point of the system evolution moves from the lower right corner of the coordinate region to the upper right corner of the coordinate region. When the NRSIs’ optimism is stronger than the FSSs’ optimism (*r*_1_>*r*_2_), the equilibrium point of the system evolution moves from the lower right corner of the coordinate region to the upward level, and the probability of the NRSIs’ positive collaborative improvement increases to about 0.4.

This conclusion is the same as that reached in the existing literature and reality, that is, due to the existence of enterprise heterogeneity [35], the optimistic combination of different intensities can promote the coordinated improvement of the quality of the new RSSC more than the optimistic combination of the same intensity. With the deepening of the optimism of functional service providers, FSSs release more positive quality signals. When the NRSIs observe the positive signals released by functional service providers, the willingness of NRSIs to participate in quality collaborative improvement increases.

#### 5.2.3. (Pessimistic, pessimistic) state analysis

**Fig 16:**
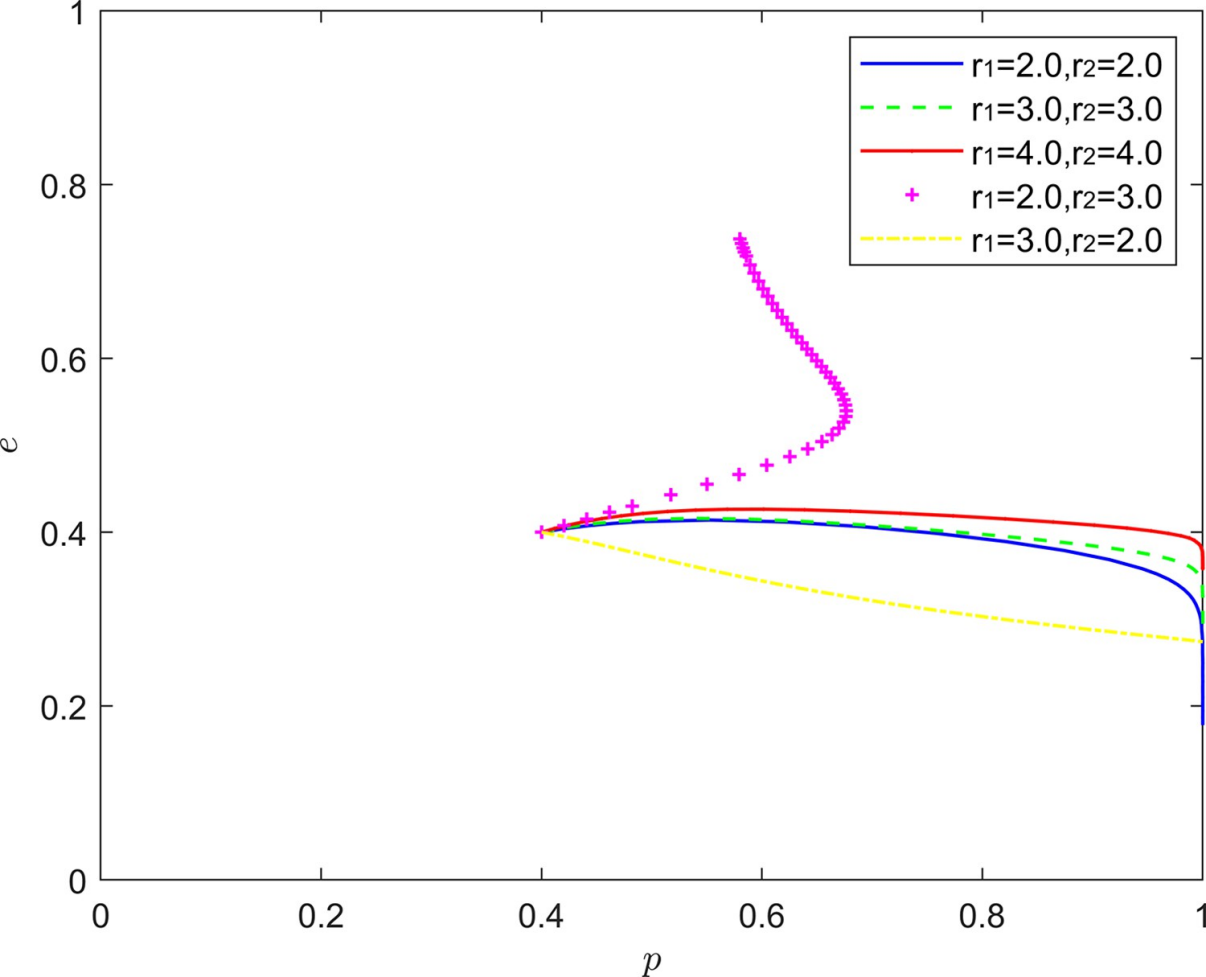
Evolution of game strategies in a (pessimistic, pessimistic) state.

Fig 16 reflects the equilibrium strategy when the FSSs are pessimistic and the NRSIs are pessimistic (*r*_1_>1,*r*_2_>1). When the pessimism of the FSSs and the NRSIs deepens in the same proportion (*r*_1_ = *r*_2_), the steady state of the system evolution does not change and the convergence rate of the system gradually slows down. When there is a difference in the intensity of pessimism between the two, a new mixed strategy Nash equilibrium point emerges in the system evolution. When the pessimism of the FSSs is stronger than the pessimism of the NRSIs (*r*_1_>*r*_2_), the equilibrium point of the system evolution is shifted upward from the lower right corner of the coordinate region, and the probability of the NRSIs’ positive collaborative improvement is increased to about 0.3. When the NRSIs’ pessimism is stronger than the FSSs’ pessimism (*r*_1_<*r*_2_). The equilibrium point of system evolution moves from the lower right corner of the coordinate region to the upper right corner of the coordinate region.

In terms of theoretical analysis, this is consistent with the conclusion in the existing literature, that is, “pessimism will prompt individuals to take positive actions” [53]. More specifically, due to the heterogeneity of enterprises, different pessimistic combinations can promote the collaborative quality improvement of the new RSSC more than the same pessimistic combination.

In terms of linkage to reality, when the NRSIs’ pessimism is deeper, the NRSIs distrust the effectiveness of the FSSs’ quality improvement more and more, and out of the pursuit of its own interests, the NRSIs choose the collaborative quality improvement more often to further safeguard the product quality and sales revenue.

#### 5.2.4. (Optimistic, pessimistic) state analysis

**Fig 17:**
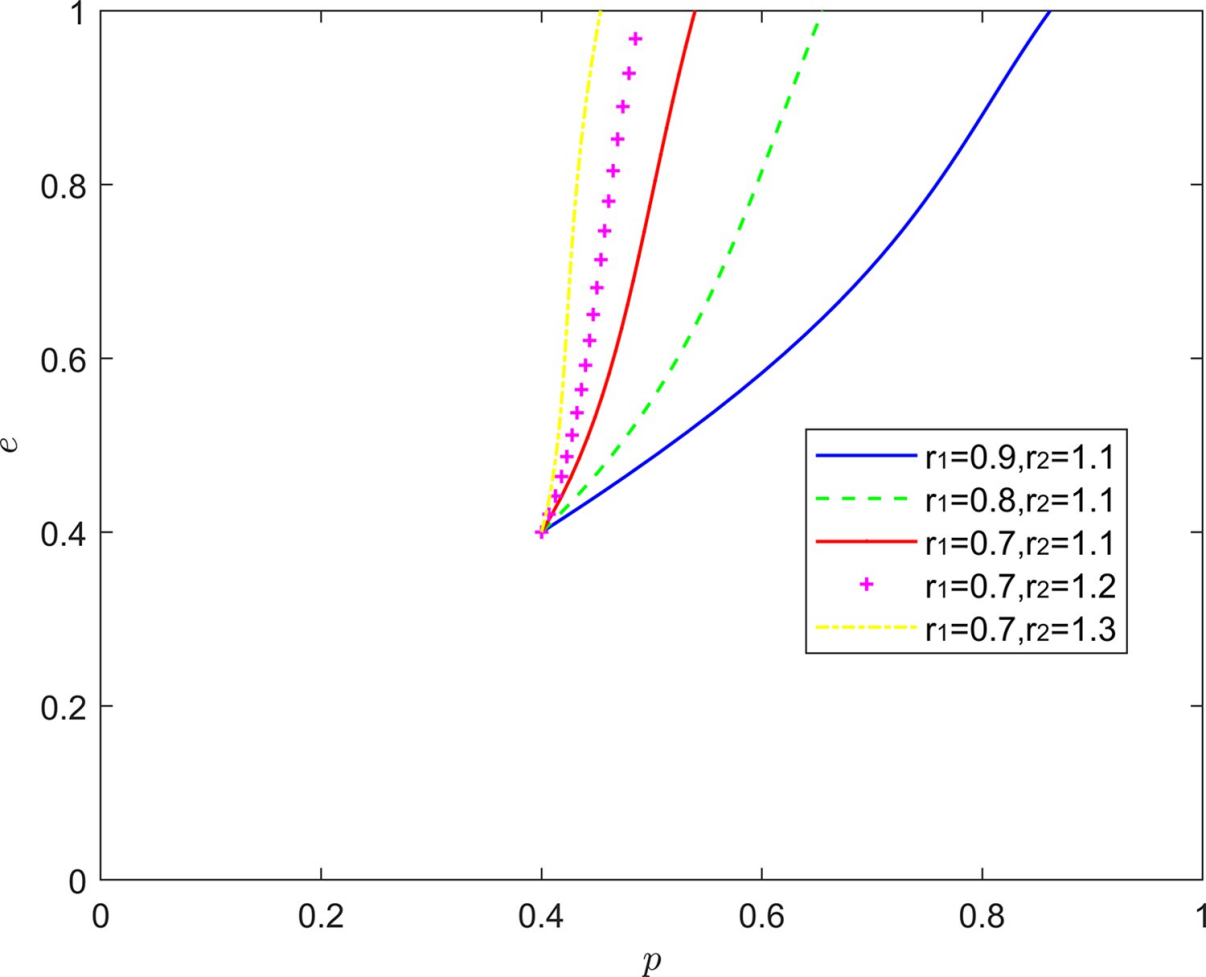
Evolution of game strategies in an (optimistic, pessimistic) state.

Fig 17 demonstrates the equilibrium strategy when the FSSs are optimistic and the NRSIs are pessimistic (*r*_1_<1,*r*_2_>1). When the FSSs are moderately optimistic and the NRSIs are moderately pessimistic, the equilibrium point of the system evolution moves from the lower right corner of the coordinate area to the upper left corner of the coordinate area. When the optimism of the FSSs or the pessimism of the NRSIs is too deep, the equilibrium point of the system evolution moves horizontally to the upper left corner of the coordinate area, and the probability of the NRSIs’ active participation in the collaborative improvement of quality decreases significantly.

In terms of theoretical analysis, these conclusions show that the interaction of emotional states leads to heterogeneity in strategy choice under different combinations of emotions [54], the influence of “optimism” on quality decision-making is not as simple as “optimism will reduce people’s willingness to choose active strategy”, but relatively complicated [55]. Therefore, moderate optimism on the part of FSSs and moderate pessimism on the part of NRSIs contribute to collaborative quality improvement in the new RSSC, whereas excessive optimism and pessimism inhibit this driving effect.

In terms of linkage to reality, the FSSs, which are optimistic about the quality improvement, release the positive quality signals while actively improving the quality, and the NRSIs, which are in a pessimistic mood, do not fully believe the quality signals transmitted by the FSSs. The pessimistic NRSIs are not fully convinced of the quality signals sent by the FSSs, and the NRSIs will participate more actively in the collaborative quality improvement the further to protect its own interests.

#### 5.2.5. (Pessimistic, optimistic) state analysis

**Fig 18:**
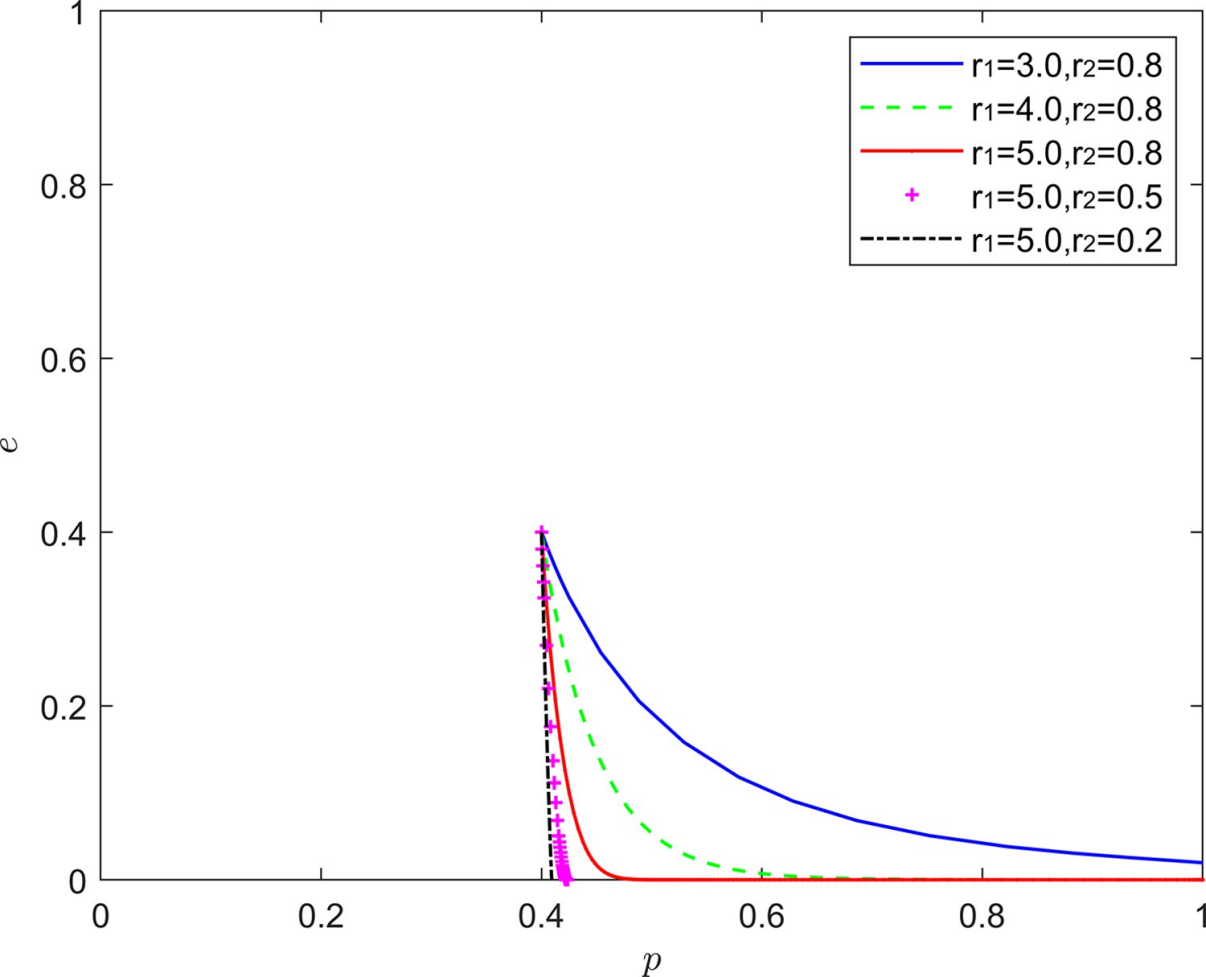
Evolution of game strategies in a (pessimistic, optimistic) state.

Fig 18 reflects the equilibrium strategy when the FSSs are pessimistic and the NRSIs are optimistic (*r*_1_>1,*r*_2_<1). The heterogeneous combination of (pessimistic, optimistic) does not change the overall trend of the system evolution, as the pessimism of the service provider and the optimism of the NRSIs deepen, the probability of the service provider choosing positive quality improvement decreases significantly, and the NRSIs’ strategy choice stabilizes at the negative collaborative improvement, and the speed of the stabilization of the strategy choice is increasing.

This is inconsistent with the conclusion that the intensity of pessimism has a greater effect on game equilibrium [56], because the combined utility of heterogeneous emotions is not a simple linear accumulation, and optimism has a greater impact on decision-making than pessimism in specific situations. In the context of this section, the heterogeneous combination of (pessimistic, optimistic) has a significant inhibitory effect on collaborative quality improvement in new RSSCs, where pessimism of FSSs reduces their willingness to invest in quality, and optimism of NRSIs causes them to over-rely on the quality inputs of the FSSs, and thus to choose inaction more often.

#### 5.2.6. (Emotional, rational) state analysis

**Fig 19:**
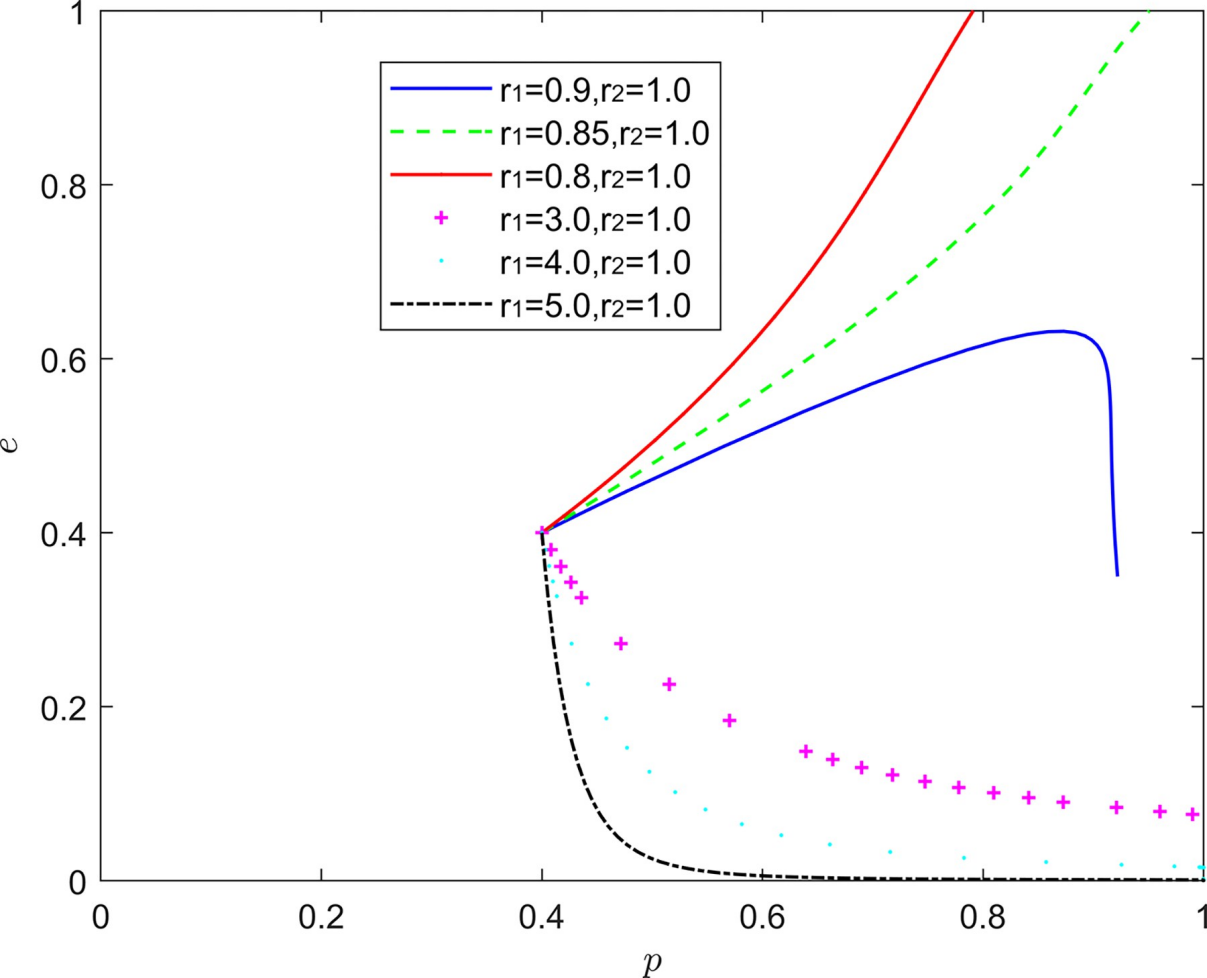
Evolution of game strategies in an (emotional, rational) state.

Fig 19 reflects the equilibrium strategy when the FSSs are emotional and the NRSIs are rational (*r*_1_≠1,*r*_2_ = 1). When the FSSs are optimistic and the NRSIs are rational (*r*_1_>1,*r*_2_ = 1), the equilibrium point of the system evolution gradually moves from the lower right corner of the coordinate region to the upper left corner of the coordinate region, but it is worth noting that the relationship between the optimism intensity of the FSSs and the positive quality improvement is nonlinear. When the FSSs are pessimistic and the NRSIs are rational (*r*_1_<1,*r*_2_ = 1), the rate at which the system evolution converges to the equilibrium of (1,0) increases significantly as the pessimism of the FSSs deepens.

The influence of emotion on quality decision-making is non-linear, and under certain circumstances, inhibition and promotion will be transformed [40]. Moderate optimism of FSSs and rationality of NRSIs help to improve the quality of the new RSSC, in which the FSSs maintain a high level of investment in quality improvement and a positive attitude, and the rational NRSIs will continue to strengthen the support and assistance to the quality improvement of the FSSs, so that they can better enjoy the positive spillover effects of the quality improvement, the intensity of optimism is not the greater (the better) or the smaller the better, but to maintain a moderate range. Therefore, it is important to be alert to the inhibitory effects of pessimism and “off-range” optimism on collaborative quality improvement.

#### 5.2.7. (Rational, emotional) state analysis

**Fig 20:**
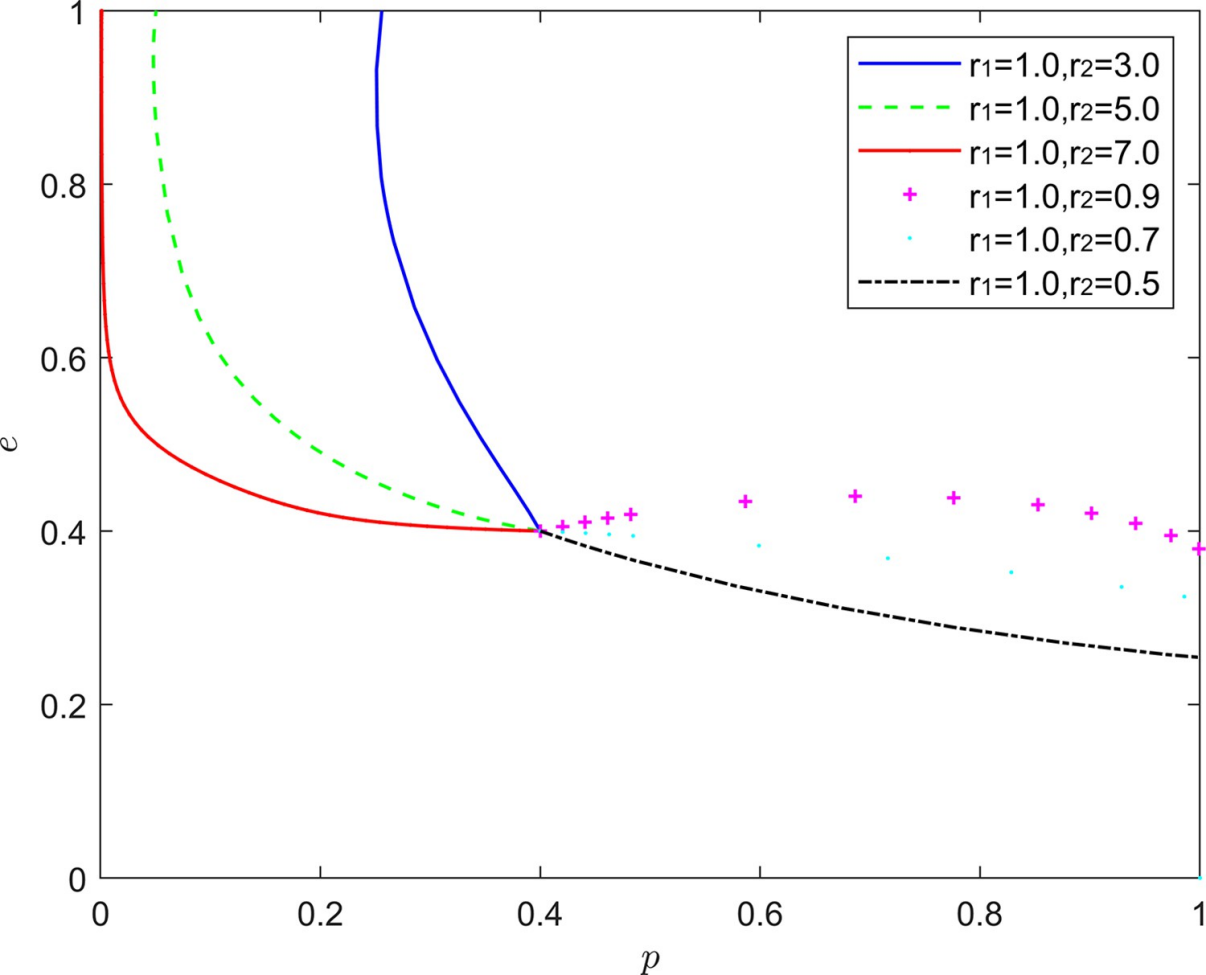
Evolution of game strategies in a (rational, emotional) states.

Fig 20 reflects the equilibrium strategy when the FSSs are rational and the NRSIs are optimistic (*r*_1_ = 1,*r*_2_≠1). When the FSSs are rational and the NRSIs are pessimistic (*r*_1_ = 1,*r*_2_>1), the equilibrium point of system evolution gradually changes from (1,0) to (0,1). When the FSSs are rational and the NRSIs are optimistic (*r*_1_ = 1,*r*_2_<1), the equilibrium point of system evolution moves upward and parallel along the coordinate axis, at which time the FSSs’ strategy choice remains unchanged, and the probability of the NRSIs’ participation in the collaborative quality improvement rises slightly.

On the one hand, the results in this part are consistent with those in the literature [57], that is, the emotions of decision makers can not affect the equilibrium of pure strategies, but will affect the equilibrium of mixed strategies. When functional service providers are optimistic, retail service integrators will move towards the strategy of “positive quality improvement”; When functional service providers are pessimistic, retail service integrators will move towards the strategy of “negative quality improvement”. On the other hand, the pessimism of retail service integrators will enhance their probability of participating in quality coordination, while rational functional service providers can choose “hitch-hiking” to save their own quality costs. Optimistic retail service integrators also have a certain probability to participate in quality collaborative improvement by amplifying the quality signals transmitted by functional service providers.

## 6. Conclusion

RDEU theory is combined with evolutionary game theory to investigate how collaborative quality improvement in new RSSC achieves the optimal quality steady state when heterogeneous emotions of decision makers are considered.

### 6.1. Key summaries

1. The effect of quality preference on the collaborative quality improvement of new RSSC is nonlinear, and this non-linear characteristic makes the quality preference of functional service providers and retail service integrators better exert their positive effect on the collaborative quality improvement of new RSSC only when they are in the moderate range. The influences of peer mechanism, feedback mechanism, and risk mechanism on the collaborative quality improvement of new RSSC are linear, and strengthening the peer reward and punishment and improving the feedback mechanism can promote the collaborative quality improvement of the new RSSC;
2. As far as the single effect of emotion on quality decision-making is concerned, that is, when only one party has emotion, the rationality of retail service integrator will promote the collaborative improvement of the quality of new RSSC more than that of functional service provider. Moreover, the influence of pessimism and optimism on quality decision-making takes the form of an inverted U-shape. When the intensity of pessimism of retail service integrators and functional service providers is too deep, the promotion of pessimism on quality decision-making will weaken; When the optimism intensity of retail service integrators and functional service providers is too deep, the inhibition of pessimism on quality decision-making will weaken;
3. As far as the combined effect of emotions on quality decision-making is concerned, that is, when both parties exhibit emotions, the combined effect of (functional service providers are optimistic, retail service integrators are pessimistic) and (functional service providers are optimistic, retail service integrators are rational) is better than other emotional combinations. In that situation, the enthusiasm of functional service providers for quality input and the positive degree of quality signals transmitted are high, and retail service integrators, with the blessing of rational or pessimistic emotions, have a higher internal driving force to participate in quality collaborative improvement.

### 6.2. Managerial implications

1. Government regulators should improve relevant laws and regulations to provide a legal basis for the practice of new retail quality management, and create a healthy developmental environment and quality atmosphere for the new RSSC through beneficial external norms. Secondly, government regulators should actively promote the transformation of external norms and constraints to internal conscious constraints, actively cultivate the quality awareness and collaborative concepts of each participating subject, and enhance the intrinsic driving force for quality improvement of the new RSSC;
2. Government regulators should take the lead in promoting the establishment of peer mechanism in the new RSSC, giving full play to the promotional role of peer incentives and the disciplinary role of peer penalties, reinforcing the supportive role of the consumer feedback mechanism for quality synergy, and reinforcing the correct perception of quality risks and enhancing the awareness of risk prevention of the main players involved, which are all measures that can contribute to the achievement of the quality homeostasis in the new retail;
3. Government regulators should promote the establishment of an open mechanism for negotiation and consultation to facilitate collaborative quality improvement in the new RSSC. Retail service integrators and functional service providers are “economic people” who pursue profit maximization, and it is difficult to achieve collaborative quality improvement only by relying on independent cooperation among enterprises. Therefore, it is necessary for government regulators to establish an open and transparent information communication and compensation mechanism, build a communication platform for retail service integrators and functional service providers, and guide them to achieve win-win results through negotiation.

### 6.3. Practical implications

1. Retail service integrators and functional service providers should continue to raise the importance of quality improvement work, establish the risk concept of “common prosperity, common loss”, and actively seek inter-enterprise cooperation with a synergistic concept, but should always be alert to the inhibitory effect of “free-rider” behavior and “non-moderate interval” quality preferences on quality improvement, to prevent the waste of resources and low input-output efficiency;
2. For functional service providers, an optimistic atmosphere should be appropriately created to actively release favorable information on quality improvement and enhance the self-confidence of functional service providers in the long-term returns of quality improvement. For retail service integrators, a rational or pessimistic atmosphere should be appropriately created, and negative quality signals should be released through various channels to strengthen the retail service integrators’ awareness of the severe situation of quality improvement in the context of new retail;
3. During the quality improvement cycle, the new RSSC node enterprises should pay attention to the emotional attitude of other node enterprises toward quality improvement, adjust their own strategies according to the actual situation, and ensure that the quality improvement is carried out in the direction of Pareto optimization. In addition, at the end of the previous cooperation cycle, retail service integrators and functional service providers should actively communicate with each other to clarify each other’s emotional attitude in the next phase of quality improvement.

### 6.4. Limitations and future research directions

Considering the significant impact of the digital drive on the new RSSC, it would be interesting to discuss the influence of new retail node enterprises’ adoption attitude towards digital technology on quality improvement. In addition, the model designed in the present work mainly discusses the behavioral evolution between FSSs and NRSIs, but the new RSSC is a complex closed-loop system with more interactions between enterprises within it. Therefore, considering the interaction relationship between enterprises in the RSSC, constructing an evolutionary game model with multi-subject participation, and investigating the influence of heterogeneous emotions on the evolutionary law of multi-subject behaviors will be a more valuable avenue of exploration in future research.

## Supporting information

S1 File (ZIP)
